# Spatiotemporal Patterns of Tumor Occurrence in Children with Intraocular Retinoblastoma

**DOI:** 10.1371/journal.pone.0132932

**Published:** 2015-07-31

**Authors:** Benjamin A. King, Carlos Parra, Yimei Li, Kathleen J. Helton, Ibrahim Qaddoumi, Matthew W. Wilson, Robert J. Ogg

**Affiliations:** 1 Department of Ophthalmology, Hamilton Eye Institute, University of Tennessee Health Sciences Center, Memphis, Tennessee, United States of America; 2 Department of Radiological Sciences, St. Jude Children’s Research Hospital, Memphis, Tennessee, United States of America; 3 Department of Biomedical Engineering, University of Memphis, Memphis, Tennessee, United States of America; 4 Department of Biostatistics, St. Jude Children’s Research Hospital, Memphis, Tennessee, United States of America; 5 Department of Oncology, St. Jude Children’s Research Hospital, Memphis, Tennessee, United States of America; 6 Department of Surgery, St. Jude Children’s Research Hospital, Memphis, Tennessee, United States of America; Universidade Federal do ABC, BRAZIL

## Abstract

**Purpose:**

To accurately map the retinal area covered by tumor in a prospectively enrolled cohort of children diagnosed with retinoblastoma.

**Methods:**

Orbital MRI in 106 consecutive retinoblastoma patients (44 bilateral) was analyzed. For MRI-visible tumors, the polar angle and angle of eccentricity of points defining tumor perimeter on the retina were determined by triangulation from images in three orthogonal planes. The centroid of the mapped area was calculated to approximate tumor origin, and the location and cumulative tumor burden were analyzed in relation to mutation type (germline vs. somatic), tumor area, and patient age at diagnosis. Location of small tumors undetected by MRI was approximated with fundoscopic images.

**Results:**

Mapping was successful for 129 tumors in 91 eyes from 67 patients (39 bilateral, 43 germline mutation). Cumulative tumor burden was highest within the macula and posterior pole and was asymmetrically higher within the inferonasal periphery. Tumor incidence was lowest in the superotemporal periphery. Tumor location varied with age at diagnosis in a complex pattern. Tumor location was concentrated in the macula and superonasal periphery in patients <5.6 months, in the inferotemporal quadrant of the posterior pole in patients 5.6-8.8 months, in the inferonasal quadrant in patients 8.8-13.2 months, and in the nasal and superotemporal periphery in patients >13.2 months. The distribution of MRI-invisible tumors was consistent with the asymmetry of mapped tumors.

**Conclusions:**

MRI-based mapping revealed a previously unrecognized pattern of retinoblastoma localization that evolves with age at diagnosis. The structured spatiotemporal distribution of tumors may provide valuable clues about cellular or molecular events associated with tumorigenesis in the developing retina.

## Introduction

Retinoblastoma is a rare cancer of the retina which presents in early childhood, typically caused by a mutation in the RB1 tumor suppressor gene. Although new treatment protocols have increased five year survival rates to over 90% [1], patients often suffer long-term visual defects secondary to irreversible retinal tissue damage [2,3] and the resulting impairment of brain structures responsible for perceiving and processing visual stimuli. Preserving vision in survivors is a central objective in medical management of retinoblastoma and clinicians often face difficult treatment decisions to balance disease control with visual outcome. A more comprehensive understanding of how retinoblastoma affects visual system function and development in the first years of life is needed to predict long term visual outcomes for retinoblastoma survivors and guide improvements in therapy.

Retinoblastoma develops and is treated during a critical period of postnatal maturation of the visual system. The tumors arise from neural retinal precursor cells that have sustained inactivating mutations in both alleles of the RB1 gene [4,5]. Most, if not all retinal neurogenesis is complete before birth [6] and tumorigenesis likely occurs during postmitotic neural differentiation and organization [7]. Tumor survival and proliferation depends on complex interactions among genetic, epigenetic and developmental factors [8,9]. The mean age of retinoblastoma diagnosis is 9 to 15 months for children with germline RB1 mutations and 23 to 36 months for children with somatic gene mutations [4,10]. Tumor may affect vision by directly disrupting the retina, however initial presentation of the tumor—its size, location and distance from the macula—appears to have only limited correlation with long term visual outcomes [11,12]. This suggests that central nervous system structures downstream of the sensory transduction apparatus in the eye are also likely involved.

We recently used functional magnetic resonance imaging (fMRI) to investigate the effects of disease and treatment on the visual system in children being treated for retinoblastoma [13]. The imaging examinations were performed during routine clinical surveillance under propofol anesthesia with photic stimulation through closed eyelids. Despite the challenging experimental conditions, functional MRI (fMRI) responses in the primary visual cortex (V1) were modulated by important clinical features, including bilateral versus unilateral tumor, macular involvement, and retinal detachment. Furthermore, the interhemispheric ratio of the volume of cortex activated in patients who had undergone unilateral enucleation reflected the expected cortical dominance in the hemisphere receiving the nasal hemiretina projection from the remaining eye. The patterns of activity within the primary visual cortex in some patients showed focal areas with no activity that appeared by visual inspection to roughly correspond to the expected location of the retinal tumor in the V1 retinotopic map. However, we were unable to systematically evaluate the precision with which the fMRI responses reflected retinotopic mapping of tumors in V1 because we did not know the precise location of tumors on the retina.

Previous studies concerning tumor location typically mapped the spatiotemporal patterns of tumor appearance to investigate clinically relevant patterns as well as topographic similarity with other retinal cell types. The studies were primarily limited to small tumors and retinal locations were approximated to sectors of the retinal surface using indirect ophthalmoscopy [14,15,16,17]. We used diagnostic magnetic resonance imaging (MRI) and imaging software to manually map the location of points on the perimeter of each tumor in retinal polar coordinates. The tumor maps were digitized and analyzed to define statistical models for the distribution of tumors over the retinal surface.

## Methods

This study included 106 consecutive retinoblastoma patients (44 bilateral) who were enrolled between 2005 and 2010 in St. Jude Children’s Research Hospital’s RET-5 protocol for the study and treatment of intra-ocular retinoblastoma (ClinicalTrials.govNCT00186888). The study was approved by the St. Jude Children’s Research Hospital Institutional Review Board (IRB), and written informed consent was obtained from each participant's parent or legal guardian according to the IRB-approved informed consent process.

Imaging was performed at the time of diagnosis of each patient with a 1.5 Tesla Siemens Symphony MRI scanner (Siemens AG, Germany) using the standard receive-only head coil. All patients were sedated during imaging with propofol (250 μg/kg/minute) under monitored anesthesia care. Our diagnostic imaging protocol comprised high resolution scans of the eye and orbit cavity including sagittal 3D T1 (TR 1920 ms, TE 2.74 ms, BW 200 Hz per pixel, FA 15 degrees, FOV 205x205 pixels, slice width 1.25 mm), axial and coronal 3D constructive–interference-in-steady-state (CISS) sequences (TR 12.72 ms, TE 6.36 ms, BW 130 Hz, FA 70 degrees, FOV 512x512 pixels). Slice width in each CISS acquisition sequence was 2.0 mm for all patients imaged prior to April 2008; slice width was 1.5 mm for all imaging sequences obtained afterward.

Image analysis was performed with Syngo Viewer software (Siemens AG, Germany) to map the tumor perimeter with respect to the two retinal coordinates: i) angle of eccentricity, ii) polar angle. The angle of eccentricity was defined as the angle between the fovea and a point on the tumor margin, measured relative to the central visual axis at the center of the lens. The polar angle was defined as the angle between the perpendicular line from the central visual axis to a point on the tumor margin, measured relative to the superior vertical meridian (defined as 0 degrees) at the fovea. All mapping was performed by the first author who was blinded to patient age.

**Fig 1:**
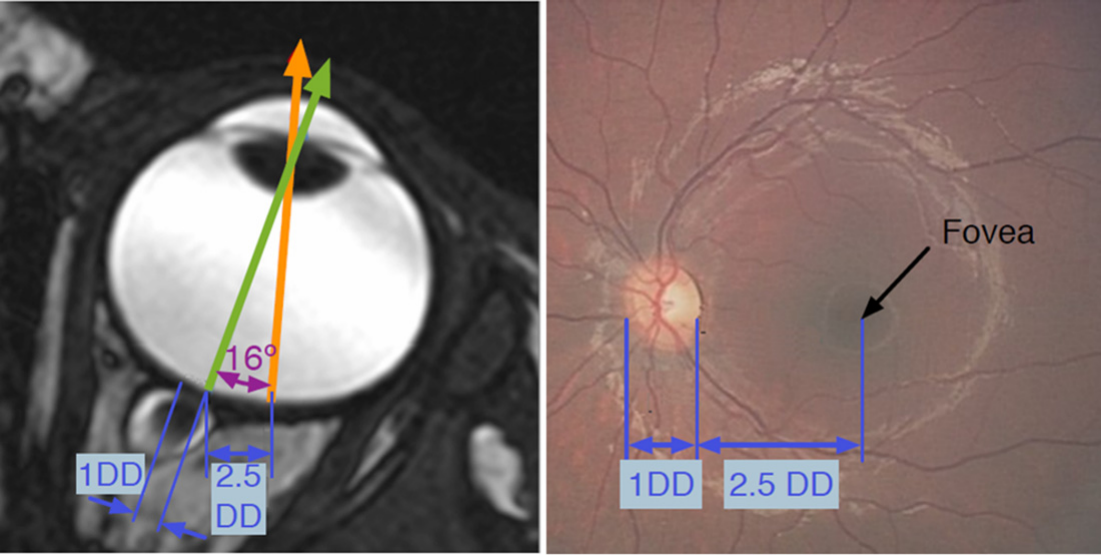
A) The fovea and central visual axis (orange arrow) were approximated on the mid-transverse axial slice by measuring exactly 2.5 disc diameters (DD) temporal to the optic nerve. B) This approximation was verified by superimposing the same measurements on RetCam fundus photography where anatomic landmarks (fovea, optic disc) can be directly visualized.

**Fig 2:**
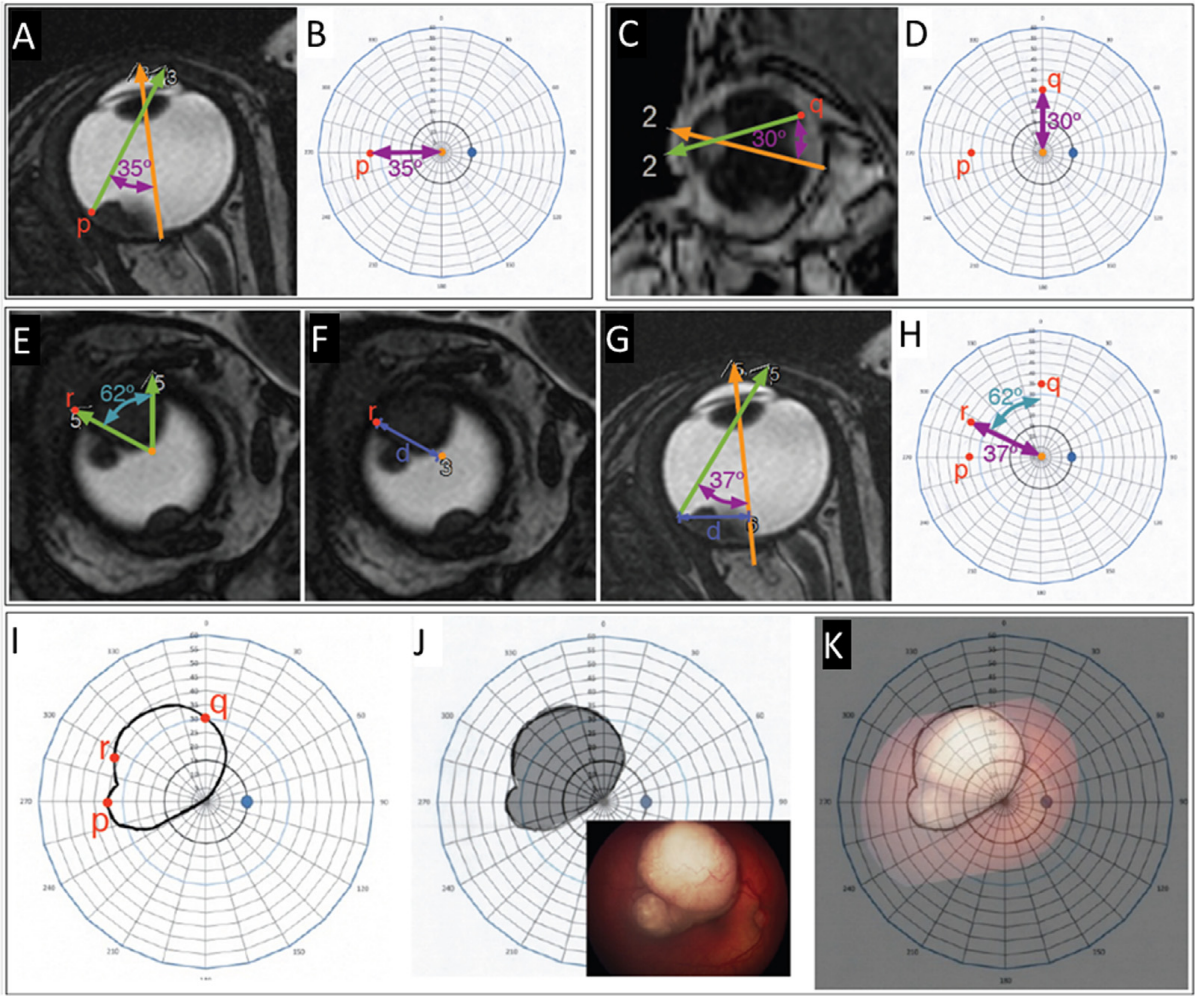
Points along the tumor margin were selected in axial (A), sagittal (C) and coronal (E) planes, and point locations were measured as angle of eccentricity relative to the central visual axis (orange arrow) or polar angle relative to the vertical meridian. Each point was then plotted on a polar coordinate graph of the retinal surface based on its measured retinal location (B, D, H). The final tumor margin was interpolated along the measured points (I, J). Funduscopic images were used to verify that the tumor graph was consistent with the actual lesion (K).

First the axial slice closest to the center of the optic disc was identified in the T2-weighted CISS sequence (Fig 1A). The fovea was approximated on this slice as being 16 degrees temporal to the optic nerve (Fig 1A). If the fovea and optic disc were visible on fundus photography at diagnosis, this approximation was refined by measuring the distance between these two landmarks in disc diameters (Fig 1B) and then superimposing the same distance on the mid-transverse MRI slice (Fig 1A). After the fovea was located, the central visual axis was defined on this slice as a line extending from the fovea through the center of the lens (Fig 1A). If the tumor was visible on the mid-transverse slice, the angle of eccentricity between the central visual axis and the margins of the tumor on the retinal horizontal meridian were measured (Fig 2A and 2B).

We cross referenced from the transverse slice to identify the sagittal slice that included the fovea and central visual axis; the eccentricity of tumor margins on the retinal vertical meridian were measured in this plane in similar fashion as before (Fig 2C). Last, a series of other points along the tumor margins were selected in cross-referenced coronal image planes and assessed for polar angle and eccentricity relative to the central visual axis. Polar angle was measured relative to the vertical meridian (Fig 2E). To measure the angle of eccentricity, the distance between the central visual axis and the selected point was measured on a coronal slice (Fig 2F). The same distance was then superimposed on the mid-transverse slice along the line of intersection with the coronal slice and the resulting angle with the central visual axis was measured (Fig 2G). No eye motion artifact was detected on any imaging obtained under sedation, and anatomic registration between cross reference planes was inspected to confirm the eye did not shift gaze position between different imaging sequences.

The measured coordinates of points along the tumor perimeter were represented on a polar coordinate graph of the retinal surface which was geometrically rendered as an azimuthal equidistant projection [18] with the origin at the fovea. The final tumor perimeter was defined by smooth interpolation between the measured points (Fig 2I). Fundus photography at diagnosis was used to verify that the tumor graph was consistent with the actual lesion (Fig 2J and 2K).

### Image Processing

Retinal tumor delineations were originally recorded on paper, using separate polar grids for left and right eyes; each polar grid is centered in the page and is enclosed by a rectangular margin. The original maps were scanned (300 pixel/inch) and coregistered for subsequent analysis. Scripts and functions for analysis of the digitized maps were written in MATLAB (The Mathworks Inc. Natick, MA) including functions in the Image Processing Toolbox. The lesion contours were marked in the digitzed maps by tracing with the Intuos4 digitizing tablet (Waco Co. Ltd., Saitama, Japan).

### Tumor Mapping

The final digitized maps of each tumor included approximately 6500 pixels in the mapped retinal area. The minimum density of pixels was 9 per degree for eccentricity and varied with polar angle from 1 pixel per degree at 5 degrees eccentricity to 12 pixels per degree at the peripheral edge of the equidistant azimuthal projection map. Pixels on or within the tumor contour were labeled as tumor. Cumulative tumor burden at each location on the retina was calculated as the number of eyes with tumor pixels at the location. Tumor area and centroid were calculated on the surface of the sphere to avoid distortions caused by the flat projection. Area was expressed as the fraction of the total mapped retinal area (i.e, to 60 degrees of visual angle). The centroid of each tumor was calculated as the center of mass of the tumor contour on the retina, without consideration of the extent of the tumor perpendicular to the retinal surface.

### Spatial Point Process Modeling

Univariate analysis of tumor centroid locations was performed with appropriate generalized linear mixed-effect models (eccentricity ≥ 0, polar angle cyclic on the interval 0°–360°). The tumor centroid distribution over the retina was also modeled as a spatial point process [19,20,21]. Based on established spatial and temporal features of retinoblastoma [14,15,16,17,22], we used a marked inhomogeneous Poisson process model (S1 Table), with marks for mutation type, tumor area quartile, and age at diagnosis quartile for each tumor. For point process modeling, the mapped polar coordinates were converted to Cartesian coordinates and the sample space was limited by a circular window with 60° radius [23]. Spatial trends in tumor density were modeled with polynomials in the retinal Cartesian coordinates. Statistical analysis and visualization was performed with R [18] including the *beeswarm*, *Cairo*, *circular*, *extracat*, *lmer*, *plotrix*, and *spatstat* packages [23–31]. Unless otherwise noted, descriptive statistics are shown in text and tables as *median*[*minimum*–*maximum*]

## Results

### Patient Summary

There were 151 affected eyes (75 right eyes, 76 left eyes) within 106 patients enrolled (Table 1). All patients with bilateral disease at diagnosis (n = 44) had germline RB1 mutations and 52 of 62 patients with unilateral disease at diagnosis had somatic RB1 mutations. The age at diagnosis (months) was significantly lower (Wilcox test, p <0.001) for patients with germline mutations (7.9 [0.4–108.3]) than with somatic mutations (21.6 [1.4–77.5]). The proportion of patients with germline mutations and unilateral disease at diagnosis was 9% of all cases and 19% of cases with germline mutation. International Classification of Retinoblastoma Groupings [32] for the sample yielded 12 eyes (7.9%) from Group A, 35 eyes (23.2%) from Group B, 18 eyes (11.9%) from Group C, 47 eyes (31.1%) from Group D and 39 eyes (25.8%) from Group E.

**Table 1:** Descriptive characteristics of the mapped patients and eyes in relation to those eligible and those unmapped.

	Eligible ^1^	p-value ^2^	Mapped ^1^	p-value ^3^	Unmapped ^1^
**Patients**					
Laterality		0.04		<0.001	
Unilateral	*62 (58)*		28 (*42*)		34 (*87*)
Bilateral	*44 (42)*		39 (*58*)		5 (*13*)
Mutation		0.11		<0.001	
Somatic	*52 (49)*		24 (*36*)		28 (72)
Germline	*54 (51)*		43 (*64*)		11 (28)
Age					
Quartiles—Mapped patients		0.02		<0.001	
(0.7)–5.6 months	19 (18)		17 (25)		2 (5)
5.6–8.8 months	18 (17)		17 (25)		1 (3)
8.8–13.2 months	19 (18)		16 (25)		2 (5)
13.2 –(65.5) months	50 (47)		17 (25)		34 (87)
Median [range] (months) ^4^	10.5 [0.4–108]	0.06	8.8 [0.7–65.5]	<0.001	26.3 [0.4–108]
**Eyes**					
Side		0.79		0.51	
Left	*76 (50)*		48 (53)		28 (47)
Right	*75 (50)*		43 (47)		32 (53)
International Classification		0.007		<0.001	
Group A	*12 (8)*		3 (*3*)		9 (15)
Group B	*35 (23)*		29 (*32*)		6 (10)
Group C	*18 (12)*		18 (*20*)		0 (0)
Group D	*47 (31)*		32 (*35*)		15 (25)
Group E	*39 (26)*		9 (10)		30 (50)

^**1**^Values indicate number of items in a category, with the number as a percentage of the group in parentheses, except under Age as per note ^4^.

^2^ Fisher’s Exact test to compare the proportions in a category between eligible and mapped groups, except under Age as per note ^4^.

^3^ Fisher’s Exact test to compare the proportions in a category between mapped and unmapped groups, except under Age as per note ^4^.

^4^ Values indicate the median and range of age at diagnosis, and p-value is for the Kolomogorov-Smirnov test to compare the age at diagnosis distributions between the groups.

**Fig 3:**
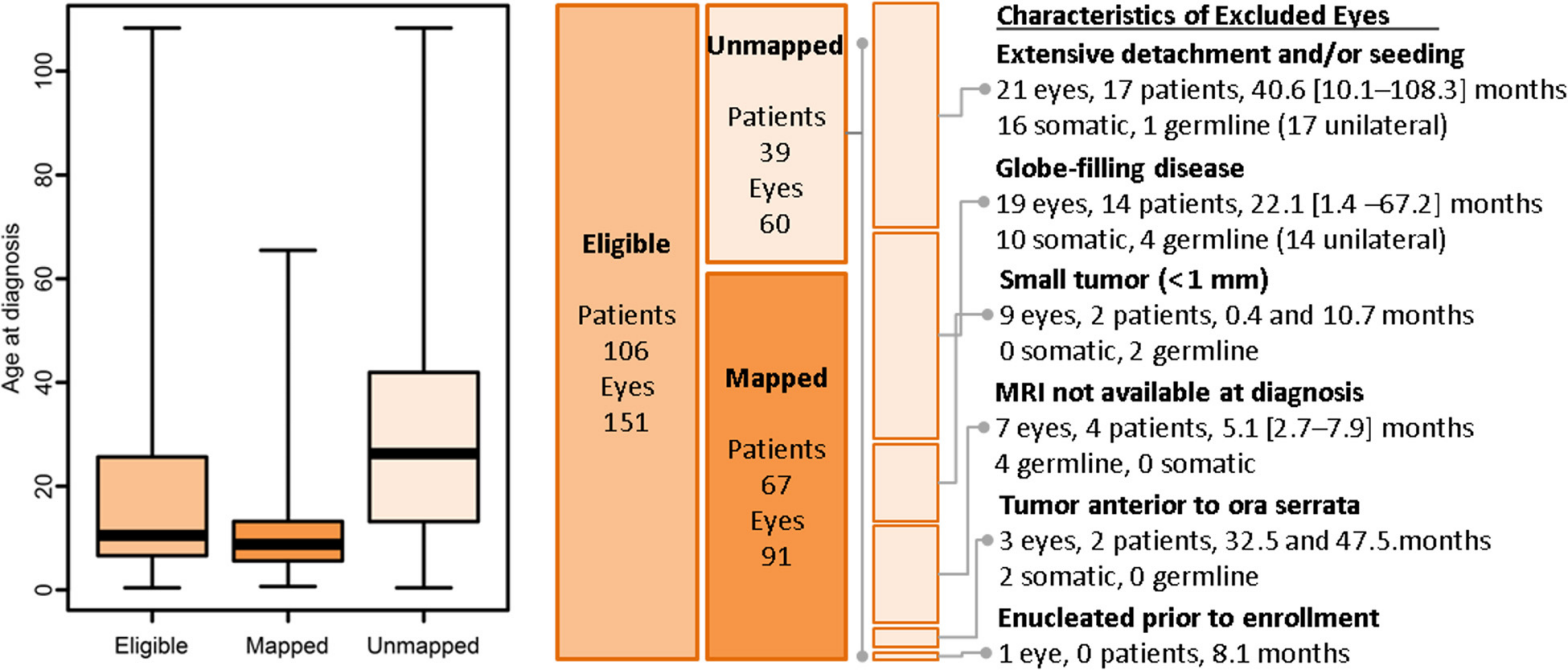
Overview of eligible, mapped, and unmapped patients and eyes. A) The distribution of age at diagnosis. Heavy bar indicates the median, vertical extent of the box indicates the interquartile range, and the whiskers indicate the full range. B) The wide bars indicate the relative proportions of the eyes in each group. The diagram at right summarizes study exclusion criteria and the number of patients and eyes excluded for each one.

**Fig 4:**
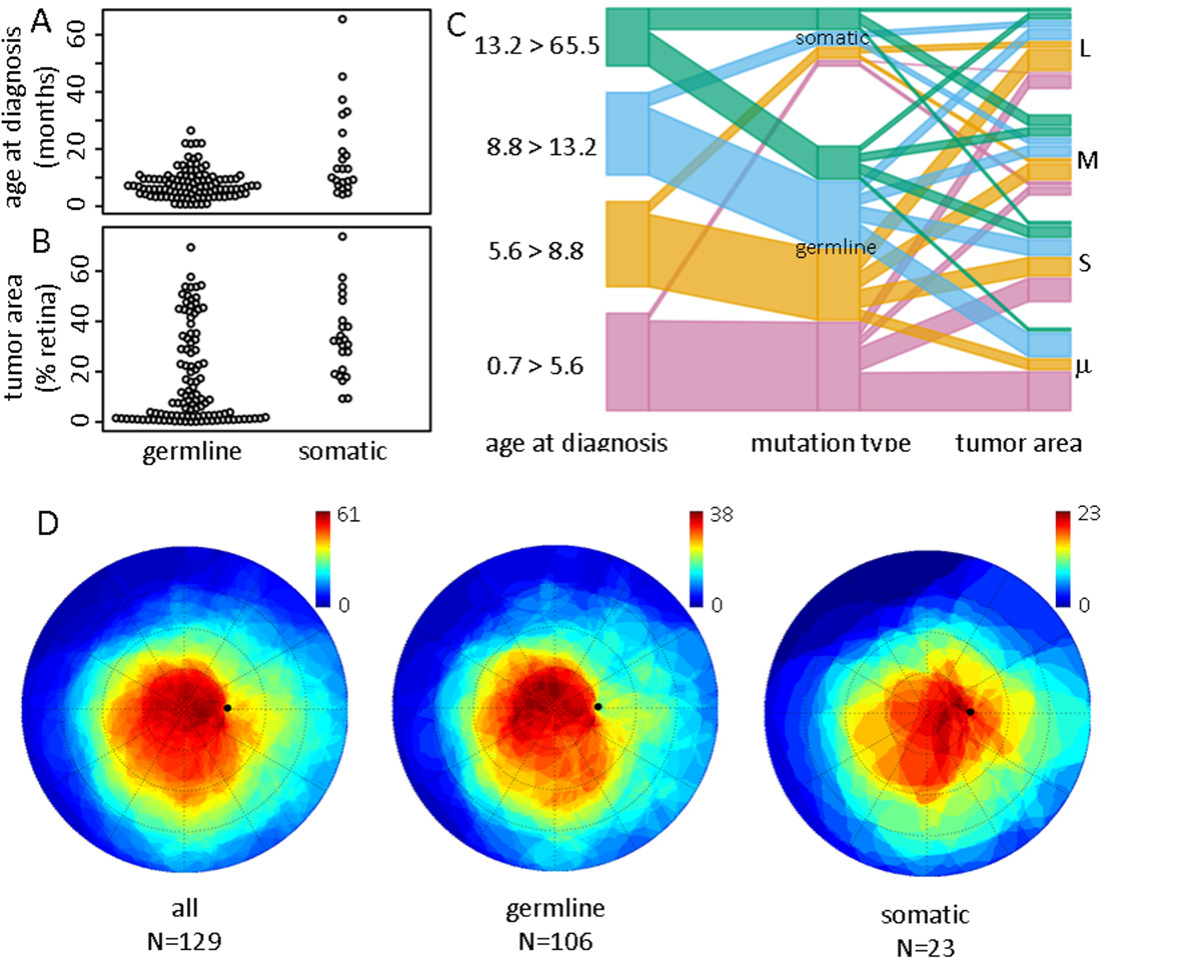
The distribution of age at diagnosis and tumor size varied between patients with germline or somatic mutations. A) Age at diagnosis and B) tumor area (expressed as % of the mapped retinal area) as a function of mutation type. C) Parallel coordinate plot illustrating the relationship among age at diagnosis, mutation type, and tumor area for all mapped tumors. Color indicates the age quartile. Tumor area quartiles: μ = smallest, S = small, M = medium, L = large. D) The cumulative tumor burden was mapped over the retina by superimposing the mapped areas of all tumors enrolled in this study. Tumors in the left eye were inverted around the nasal-temporal axis to preserve spatial symmetry with right eye tumors. The color legend indicates the number of tumors which were mapped over a specific point.

Tumors were successfully mapped in 91 eyes (43 right eyes, 48 left eyes) from 67 patients (43 germline). These included 3 eyes (3.2%) from Group A, 29 eyes (31.9%) from Group B, 18 eyes (19.8%) from Group C, 32 eyes (35.1%) from Group D and 9 eyes (9.8%) from Group E. The characteristics of excluded eyes are summarized in Table 1 and Fig 3. Most exclusions were for extensive disease that rendered the mapping unfeasible or uninformative, therefore the excluded group was biased toward larger tumor area (Wilcox test, W = 656, p <0.001) in older patients (Wilcox test, W = 598, p <0.001) with somatic mutations (Fisher test, OR = 4.5, p <0.001). For the mapped group, age at diagnosis and tumor size were analyzed as factors after quartile split and Fig 4C illustrates the multivariate relationships among age at diagnosis, mutation type, and tumor area.

### Mapping Tumor Burden

We calculated the cumulative incidence of tumor at each location on the mapped retinal surface to summarize the spatial distribution of tumors across the group. The distribution of tumors was similar in right and left eyes (Fisher test, OR = 1.3, p = 0.51), so we inverted the nasal-temporal axis of tumor maps for the left eyes and rendered the overall density on the right eye polar plot (Fig 4D). Consistent with previous studies of the distribution of retinoblastoma on the retina, tumor count was highest over the macula and posterior pole, and decreased progressively with increasing eccentricity. Unexpectedly, tumor distribution was asymmetric in the peripheral retina, with apparently higher cumulative burden in the nasal and inferonasal retina than in the temporal retina at equal eccentricity.

The general features of tumor distribution were apparent in patients with both germline and somatic RB1 mutations when analyzed in separate subsets (Fig 4D). Compared to the somatic mutation group, tumor incidence in patients with germline mutations was more heavily concentrated within the temporal-posterior pole. The cumulative tumor burden outside the posterior pole was lower in patients with germline mutations than in patients with somatic mutations. The density of tumors at a retinal location reflects the distribution of both the point origin and the size of tumors across the cohort. As will be detailed below, we subsequently mapped the cumulative tumor burden separately within each age quartile to characterize the pattern of disease with increasing age at diagnosis (Fig 7C). The distribution of tumor burden varied strongly by age quartile and reflected a previously unrecognized regularity in the distribution of retinoblastoma over the retina.

### Spatial and temporal patterns of tumor occurrence on the retina

**Fig 5:**
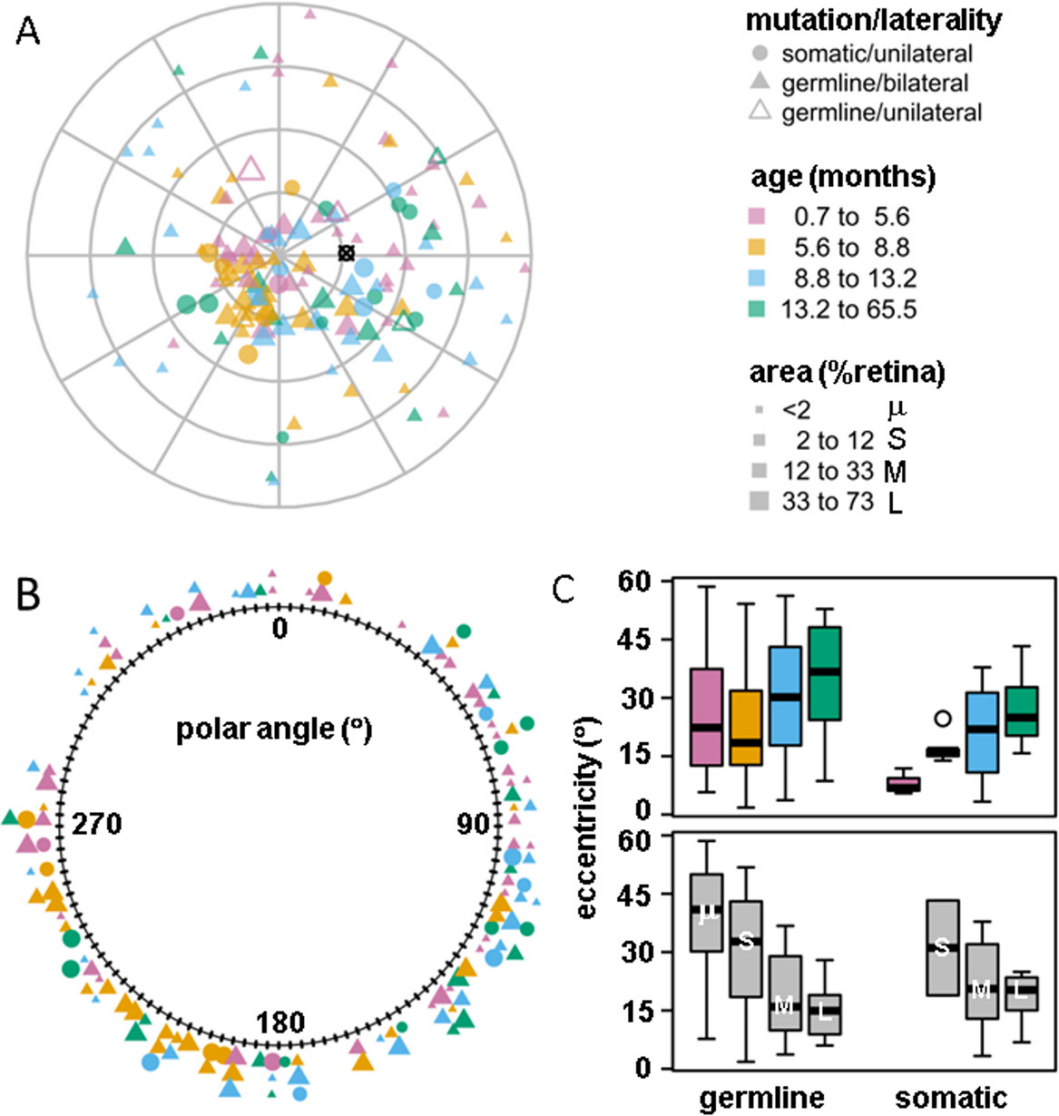
A) Spatial distribution of tumor centroids rendered in the azimuthal-equidistant projection. Plotting symbol color indicates age at diagnosis quartile, and symbol size indicates tumor area quartile, and symbol shape indicates mutation type and disease laterality. B) Distribution of all tumor centroids with respect to polar angle. The optic disk was located at 88 degrees. C) Box-and-whisker plots for tumor centroid eccentricity as a function of mutation type (germline vs. somatic) by age at diagnosis (quartiles, as indicated by color) and by tumor area (quartiles with μ = smallest, S = small, M = medium, L = large).

To further characterize the unexpected asymmetry of the cumulative tumor incidence, we analyzed the distribution of tumor centroids over the retina. Consistent with the overall tumor density, tumor centroids were inhomogeneously distributed across the retinal surface (Fig 5). Centroid eccentricity was associated with tumor area (F(3,60) = 11.8, p<<0.0001) and age at diagnosis(F(3,60) = 6.4, p<0.0007), with no significant interaction between these factors, and the within-subject variance of tumor area was significant (F(3,59) = 13.2, p<0.0001). Eccentricity did not vary significantly with mutation type. Post-hoc comparison showed that eccentricity differed between all levels of tumor area (Tukey honest significant differences, p-adjusted < 0.002), except for the two largest (L-M) and the two smallest (S-μ). For age at diagnosis, eccentricity differed between the oldest and youngest quartiles and between the oldest and second youngest quartiles ((Tukey honest significant differences, p-adjusted < 0.007) (Fig 5C). The centroid polar angle varied with age (F(3,63) = 4.0, p = 0.01), but not with mutation type or tumor area (Fig 5B). Polar angle of the second age quartile differed from all other age quartiles in post-hoc comparison (Tukey honest significant differences, p-adjusted = 0.04).

**Fig 6:**
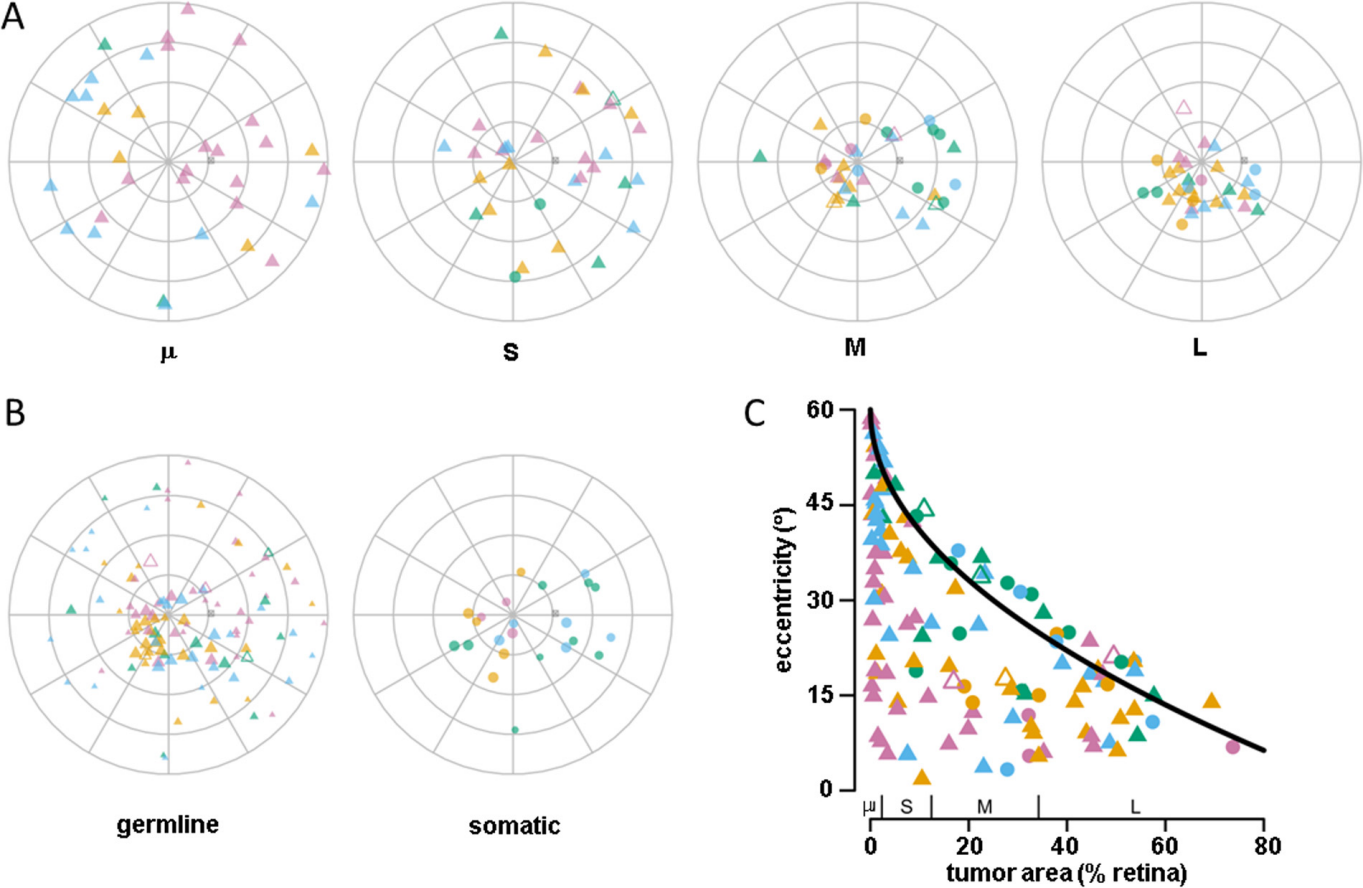
The tumor centroid distribution rendered in the azimuthal-equidistant projection by (A) tumor area quartiles and (B) mutation type. C) Scatter plot of tumor centroid eccentricity vs. tumor area. The range of each tumor area quartile is indicated on the x-axis. The black curve shows the approximate maximum eccentricity for a tumor of a given size (see text). Plotting symbol color indicates age at diagnosis quartile (magenta, yellow, blue, green) and shape indicates mutation type and disease laterality.

The distribution of tumor centroids varied with tumor area (Figs 5 and 6). Tumor foci in the smallest size quartile were the most uniformly distributed and showed the most uniform distribution of tumor incidence between the central and peripheral retina. In each subsequently larger quartile, the distribution of tumors was increasingly concentrated in different parts of the retina. We note that the distribution of tumors with respect to tumor area (Fig 6A) and also with respect to mutation type (Fig 6B) show some evidence of symmetry around the optic disk. The concentration of larger tumors near the posterior pole may be biased in our analysis because we estimated tumor origin from the centroid. To estimate the potential bias, we calculated the approximate maximum eccentricity of the tumor center as a function of tumor area, assuming a circular shape (~(1-area^1/2^)) just touching the limit of the mapped retina. The analysis showed that only 25% of all tumors were located near the physical limit such that the tumor eccentricity was constrained by the tumor size, and only 30% of tumors even in the largest size quartile were near the limit (Fig 6C). This suggests that the inhomogeneous distribution of tumor locations with respect to size reflects features of tumorigenesis and eye development, rather than an artifact of estimating tumor origin from tumor centroid (i.e., tumors are large because they are central, not central because they are large). We note that there were no remarkable differences in patterns of tumor occurrence between eyes assigned to different International Classification groups beyond the Classification-related effects of age at diagnosis and tumor area already described.

**Fig 7:**
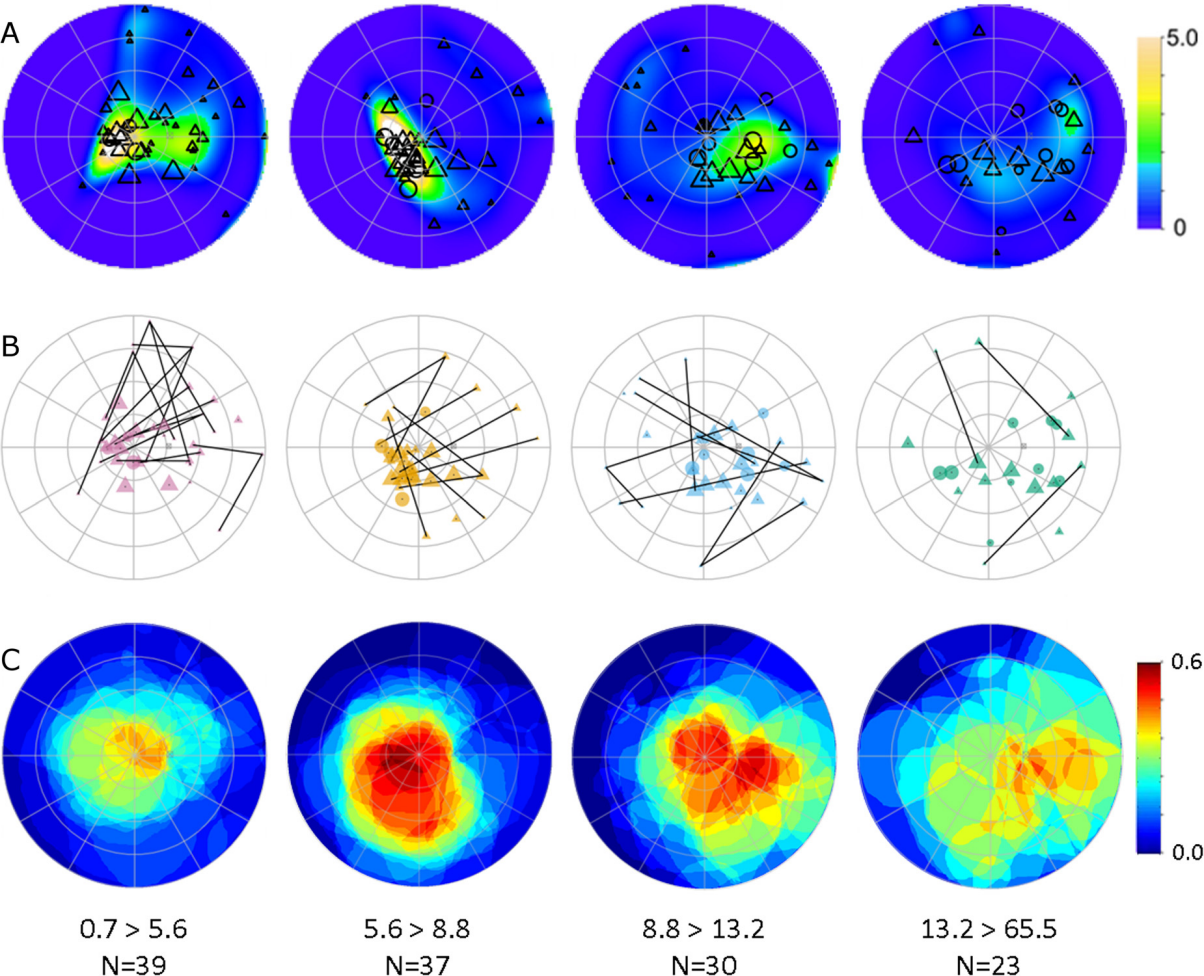
Tumor centroid distribution in each age quartile. A) Spatial point process model with significant clustering of tumor centroids in different parts of the retina as a function of age at diagnosis. The color code indicates the density of tumor centroids relative to the density expected for a completely random spatial distribution of the same number of tumors on the mapped retinal surface. B) Polar plot of tumor centroids in each age quartile with lines connecting multiple tumors within an eye. C) Mapped tumors within each age quartile are superimposed over one another to map the cumulative tumor burden for each quartile. The color code indicates the fraction of the tumors in each age quartile that overlap at a given location.

Tumor centroids were clustered in different retinal areas depending on the age at diagnosis (Fig 7). Retinoblastoma diagnosed at ages less than 5.6 months occurred most frequently in the macula and superonasal periphery. Tumors diagnosed between 5.6 and 8.8 months of age were most likely to occur within the inferotemporal quadrant of the posterior pole, immediately adjacent to the macula. Between 8.8 and 13.2 months, most tumors were concentrated within the inferonasal quadrant of the posterior pole. After 13.2 months tumor incidence continued to advance nasally and anteriorly but was more diffusely scattered over the retina compared to the more clustered pattern in younger patients. It is noteworthy that age-related clustering was similar in patients with germline or somatic mutations.

To elaborate our analysis of tumor location, we used spatial point process models to characterize the apparent age-related inhomogeneity of tumorigenesis over the retinal surface [19,20,21]. The second order moments (i.e., pairwise relationships) [24] of the overall tumor distribution showed that there was significant clustering compared to so-called complete spatial randomness, even after allowing for non-stationary probability density over the retina (S1 Fig). Note that the sophisticated tools for spatial statistical analysis in ‘spatstat’ package are based on Euclidean distances, rather than on great circle distances most appropriate for the retinal surface. We compared the pairwise nearest neighbor great circle and Euclidean distances for all tumors to estimate the potential impact of this distance error on the modeling results. Of course, the actual nearest neighbor distances (great circle) were always less than or equal to the Euclidean distances (S2 Fig), but the differences were not significant (Wilcox test, W = 10857, p = 0.27). Furthermore, any systematic effect of the distance error would tend to diminish the clustering pattern that we observed. Consistent with our univariate analysis of eccentricity and polar angle (Fig 5B and 5C), spatial point process models based on the Euclidean distance showed that the inhomogeneous Poisson density varied with mutation type, tumor area, and age at diagnosis (Fig 7A). These factors are not independent (Fig 4D) and the variations in tumor density are consistent with the reported central-to-peripheral pattern of retinoblastoma incidence [14, 15, 17] and concentration of tumors along the horizontal meridian [16]. However, the complex variation in tumor location with age at diagnosis has not been reported.

Subsequent analysis of the pattern of localization of multiple tumors within an eye revealed additional evidence of age-related clustering in the spatial distribution of tumors over the retina. The age-related variation in tumor density (Fig 7A) reflects clustering of both posterior and anterior tumors. The pattern of lines connecting multiple tumors within an eye (Fig 7B) shows that the age-related clustering was driven in part by the concentration of larger central tumors and in part by the concentration of smaller peripheral tumors in different locations. There was a surprising regularity of the spatial relationship between central and peripheral tumors within an eye that was consistent with the overall localization within each age group (see also individual patient maps in S4 Fig). It appeared that the distance between tumors within an eye was greater than the typical distance between tumors, even within an age quartile. Permutation testing with random sampling from the full data set to match the observed subset of multiple tumors confirmed that nearest neighbor distances among multiple tumors within in an eye was significantly greater than the nearest neighbor distances for comparable samples from the overall spatial pattern across patients (S2B Fig). However, the marginal separation between tumors within an eye *increased* with the distance between tumor centroids, demonstrating that the nearest neighbor distances among multiple tumors within an eye were not driven by contact or crowding between the tumors (S2C and S2D Fig).

### Distribution of Unmapped Small Tumors within Mapped Eyes

**Fig 8:**
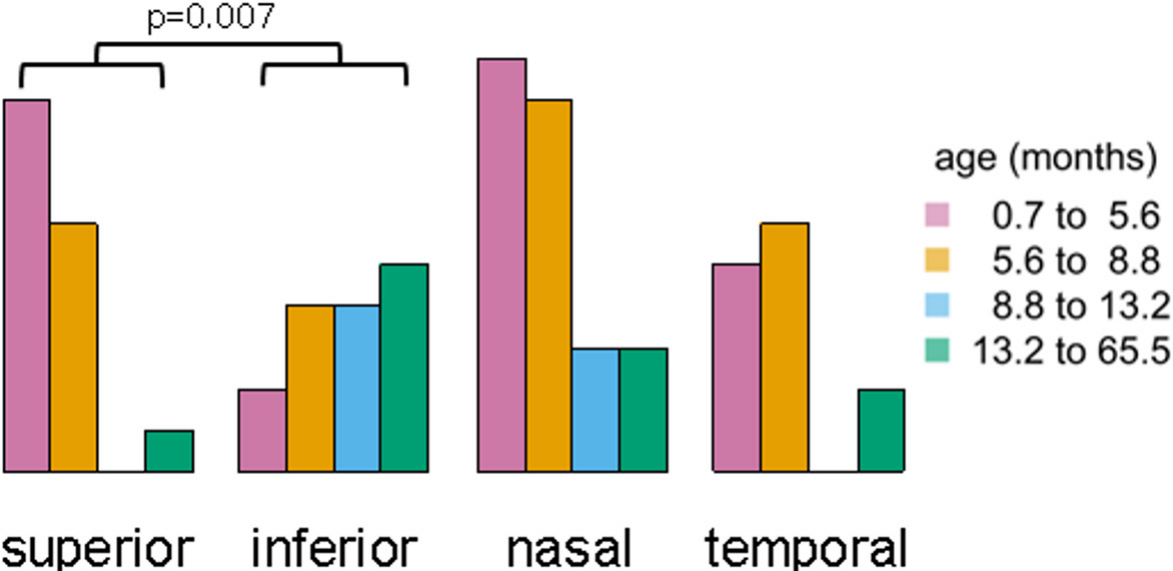
Distribution of approximated locations for all 50 unmapped tumors with respect to each patient age quartile. With increasing age at diagnosis, there was a significant increase in tumor frequency in the inferior quadrants while the number of tumors noted in superior quadrants decreased. No significant effect was noted in variation of tumor occurrence between nasal and temporal quadrants with respect to age.

Of the 147 tumors identified clinically in mapped patients with bilateral disease, 50 were too small (<1–2 mm) for detection with diagnostic MRI and were not included in the above analysis. The locations of the small tumors reported from fundoscopy were as follows: 42 anterior to the equator with 13 infranasal, 9 supranasal, 6 anterior nasal meridian, 7 supratemporal, 4 infratemporal and 3 anterior temporal meridian; and 8 tumors were posterior to the equator with 3 infranasal, 1 supranasal, 1 supratemporal, 1 infratemporal, and 2 in the macula. The distribution of the unmapped tumors varied over the age quartiles (Fig 8), and the effect was significant for the superior/inferior proportions (Fisher exact test, p = 0.008). Plots of the small tumors by quadrant along with the mapped tumors (S3 Fig) show additional evidence that the distribution of the unmapped was generally consistent with the pattern apparent in the mapped tumors.

## Discussion

We used diagnostic MRI to map the perimeter of primary tumors on the surface of the retina in retinoblastoma patients at the time of diagnosis. MRI is not conventionally used to characterize intraocular disease because clinical fundoscopy is effective, and more sensitive for detecting small lesions (e.g., Group A). However, spatial metrics can only be roughly quantified with standard clinical fundoscopy, and in eyes with more extensive disease, anatomic points of reference such as the fovea and optic nerve are obscured by tumor growth making it impossible to quantify tumor margin location using fundus photography alone. With MRI, retinal coordinates can be objectively measured in a consistent retinal coordinate space, and the full extent of ocular anatomy can be evaluated regardless of tumor size. The MRI-based tumor maps we produced provide information about the tumor burden in each patient that is essential for evaluation and interpretation of fMRI findings that we have reported in this patient population [13]. In addition, the systematic mapping in this cohort revealed previously unrecognized patterns of age-related tumor localization over the surface of the retina.

The spatial and temporal patterns we report are consistent with well-established patterns for intraocular retinoblastoma [14–17]. The previous studies were based on fundus drawings and limited to small tumors to approximate the tumor point of origin. There is a central-to-peripheral progression of retinoblastoma location with increasing age at diagnosis [14, 15, 17]. Abramson et al. categorized tumors in patients with bilateral disease into one of four concentric zones around the fovea and noted that average age at diagnosis increased in each subsequently more peripheral zone. Posterior pole tumors were diagnosed at the youngest age and subsequent tumors were never detected within the macula [14, 15]. Brinkert, et al. measured the distance between the macula and the tumor center and detected a similar distribution [17]. This central-to-peripheral trend is apparent in our data (Figs 5C and 7A), with median eccentricity increasing in the later age quartiles for tumors arising from both germline and somatic mutations.

Earlier studies also demonstrated that retinoblastoma is more frequently located in the nasal hemiretina. Brinkert, et al. did not address the azimuthal distribution of tumors [17]. Munier, et al. analyzed the distribution of tumors in 12 wedge-shaped sectors of the retinal surface corresponding to the hours of a clock face [16]. Their analysis included patients with bilateral and unilateral disease, and demonstrated an asymmetric distribution (nasal > temporal) and a larger fraction of tumors near the horizontal meridian than the vertical meridian. Abramson, et al. compared the number of tumors by hemisphere (superior vs. inferior, temporal vs nasal) and found no significant differences [14], but we note that the reported hemispheric ratios of tumor counts were similar to the asymmetric ratios reported by Munier [16]. Consistent with these earlier studies, the overall distribution of cumulative tumor density (Fig 4D) and tumor centroids (Fig 5A) within our cohort—regardless of mutation type, tumor size or age at diagnosis—was concentrated within the inferior posterior pole. Peripherally, tumor occurrence was asymmetrically higher in the nasal versus temporal hemiretina.

Several factors likely account for the relatively high fraction of central tumors in our study. The earlier studies primarily comprised small tumors and had a lower threshold for exclusion of larger lesions. Among bilateral patients, Abramson et al. excluded 80% of fellow eyes with more advanced disease [14] whereas only 23% were excluded in our study. According to our data, centroids of the largest tumors are localized mostly within the inferior half of the posterior pole; only one tumor centroid was mapped above the horizontal meridian in the largest tumor quartile. Tumors in the smallest quartile were most widely and homogeneously distributed over the retina—a pattern more consistent with previous studies that presumably excluded many centrally occurring lesions as they tend to be too large once diagnosed to localize with indirect ophthalmoscopy. In order to understand how tumor occurrence trends change during the first years of life, it is necessary to account for these more advanced lesions which arise earlier and more centrally according to our data.

We also note that an additional 18% of fellow eyes were excluded in our study with Group A disease undetectable with MRI. Our analysis of similar small unmapped tumors detected among the eyes with mapped tumors showed that 84% of these lesions were anterior to the equator. Finally, unlike previous studies we did not map subsequent tumor occurrence after the initial diagnosis. New tumors are more prone to occur peripherally [14, 15] and their exclusion along with MRI-undetectable disease likely increases the observed difference between central versus peripheral tumor occurrence.

Our MRI-based tumor mapping and analysis of digitized tumor maps revealed surprising regularities in the location of tumors as a function of age at diagnosis. Tumors diagnosed within each age quartile were concentrated in different retinal areas, and the age-related localization was much more complex than central-to-peripheral progression and nasal predominance previously reported [14–17]. The distribution within each quartile reflects concentration of both central and peripheral tumors. The concentration of more central tumors progressed by age quartiles in a roughly circumfoveal trajectory from superotemporal to inferotemporal to inferonasal to nasal meridian, with an increasing average eccentricity. The peripheral tumors were essentially split at the median, with tumors from the youngest half of patients in the nasal hemisphere and the oldest half of patients in the temporal hemisphere. The most peripheral tumors in each quartile were in eyes that included more central tumors, and the peripheral tumors had a remarkably regular relationship to the central tumors in the same eye. The age-related localization was similar for tumors arising from germline and from somatic mutations, and age-related separation of tumor locations is apparent within each tumor area quartile.

The robust and regular age-related pattern of tumor localization that we identified may provide important clues to retinoblastoma genesis. The asymmetry of this pattern may arise from the similarly asymmetric topographic distribution of the retinoblastoma cell of origin, if such a cell exists. The topography of disease incidence that we observed was consistent with the topography of multiple retinal cell populations. Some evidence suggests that the cone photoreceptor precursor is a candidate cell of origin, based on tumor expression of cone-specific photopigments and phototransduction proteins [33,34]. A prior study of disease topography supported this hypothesis based on the similarity of the nasally-skewed, central-to-peripheral gradient distribution of tumors to that of cone photoreceptors [16]. However, the topography of amacrine cells (and other retinal neural cells) is similar to that of cone cells [35] and a recent study found tumor phenotypes more consistent with amacrine and horizontal cells [36]. This was corroborated by an investigation with spectral domain optical coherence tomography (SD-OCT) which found tumors were more likely to originate within the amacrine cell layer than in the outer segment [37].

Many prenatal and postnatal events in the developing retina may render some areas more susceptible to undergoing tumorigenesis. Early in development, patterning events direct the differentiation and distribution of certain cell populations along the dorso-ventral or nasal-temporal axis of the globe by inducing the expression of a specific combination of transcription factors [38, 39, 40]. Some of these transcription factors are known to actively promote tumor growth and inhibit apoptosis in cultured retinoblastoma cell lines [41, 42]. A similar mechanism of action in vivo may contribute to the nasally skewed topography of retinoblastoma. The asymmetric expansion of the retinal vascular system in utero may also affect the pattern of tumorigenesis. The nasal periphery is perfused several weeks prior to the temporal periphery [43] and the topographic progression of both vasculogenesis and angiogenesis differs significantly between these areas [44]. Vascular supply is critical for retinoblastoma progression [45, 46] and its asymmetric development in the retina may also contribute to the variation in tumor incidence and time of onset between the nasal and temporal periphery.

The evolution of tumor location with age that we observed is similar to patterns for neurogenesis and cell differentiation in the retina, with progression in a circumferential manner around the posterior pole while expanding outward towards the periphery [47,48,49]. The similarity of the spatial trends in tumor incidence and retinal development is consistent with reported mechanistic correlation between tumorigenesis and developmental expression of signals required for tumor cell survival and proliferation [8,9,50,51]. These genetic and epigenetic events occur within limited spatiotemporal intervals during retinal maturation [8], and therefore the complex spatiotemporal pattern of tumor occurrence may help to identify critical events that cause malignant transformation of retinoblasts with inactivating RB1 mutations [4].

## Conclusion

We demonstrated a distinctive pattern of topographic variation in retinoblastoma incidence that varied with the age of tumor diagnosis. The spatiotemporal distribution of tumor locations likely reflects regional developmental events in the young retina that facilitate cell survival and proliferation after the requisite inactivating mutation in RB1 is acquired. Identification of biological correlates of the spatial and temporal patterns we observed may help to clarify the mechanisms of retinoblastoma genesis. Understanding the spatiotemporal evolution of disease incidence may also help clinicians anticipate new intraocular lesions in germline retinoblastoma and their resulting visual field defects. Ultimately we will use this detailed assessment of retinal topography to characterize the relationship between tumor manifestation in the eye and resulting functional deficits in the visual system as quantified by functional MRI (fMRI).

## Supporting Information

S1 Fig Spatial point process analysis of the distribution of tumor centroids on the retina. Ripley’s K statistic for the nearest neighbor distance showed that the tumors centroids (black curve) were more clustered than would be expected for a spatially random distribution (gray curve).
(PDF)

S2 Fig A) Euclidian nearest neighbor distance in the azmuthal equidistant projection was greater than or equal to the actual great circle distance on the retinal surface, but the overall difference was not significant. B) The distribution of nearest neighbors distances for the subset of tumors in eyes containing multiple tumors (histogram and density curve (thick line) was significantly different from the distribution for similar samples from the full set of tumors estimated by permutation testing (thin line), with larger distances among tumors within an eye. C) The margin between tumors within an eye was estimated as the shortest distance between the boundaries of the tumor for comparison with the distance between the centroids of the tumors. D) The margin/distance ratio increased with distance, and the minimum ratio (~0.4) shows that the observed distribution of multiple tumors within an eye was not driven by close contact or crowding.
(PDF)

S3 Fig Tumor centroid plots by individual patient, shown in azimuthal equidistant projection corresponding to a right eye. The plots include all mapped tumors and also the small tumors that were unmapped, but were located by quadrant from clinical fundoscopy reports. Tumors from right eyes are indicated with a + sign, tumors from left eyes are unmarked. Patient age at diagnosis is indicated above each plot, and the plots are presented in order of increasing age.
(PDF)

S1 Table Poisson Point Process Models for distribution of tumor centroids marked by age at diagnosis quartile with polynomial covariates. (PDF)
