# Supplementary material for: Spatiotemporal Patterns of Tumor Occurrence in Children with Intraocular Retinoblastoma
Source: PLoS One. 2015 Jul 31;10(7):e0132932. doi: 10.1371/journal.pone.0132932 (PMC4521796; doi:10.1371/journal.pone.0132932)
Supplement: S1 Fig — Ripley’s K statistic for the nearest neighbor distance showed that the tumors centroids (black curve) were more clustered than would be expected for a spatially random distribution (gray curve). (PDF) [file pone.0132932.s001.pdf]

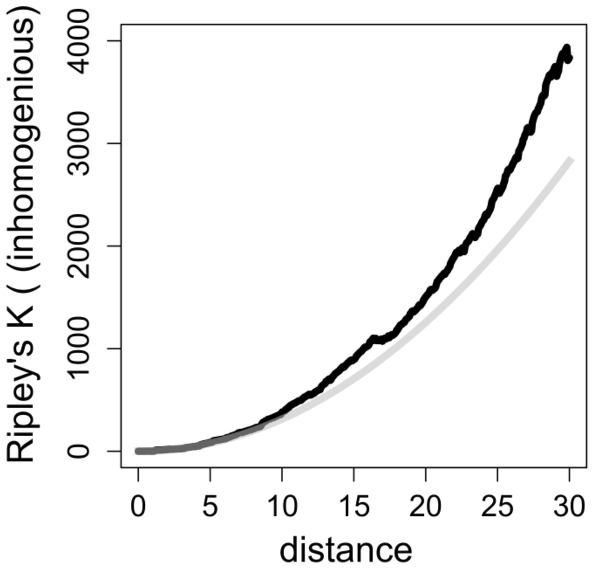

S1 Fig. Spatial point process analysis of the distribution of tumor centroids on the retina. Ripley's K statistic for the nearest neighbor distance showed that the tumors centroids (black curve) were more clustered than would be expected for a spatially random distribution (gray curve).
