# Supplementary material for: Spatiotemporal Patterns of Tumor Occurrence in Children with Intraocular Retinoblastoma
Source: PLoS One. 2015 Jul 31;10(7):e0132932. doi: 10.1371/journal.pone.0132932 (PMC4521796; doi:10.1371/journal.pone.0132932)
Supplement: S2 Fig — A) Euclidian nearest neighbor distance in the azmuthal equidistant projection was greater than or equal to the actual great circle distance on the retinal surface, but the overall difference was not significant. B) The distribution of nearest neighbors distances for the subset of tumors in eyes containing multiple tumors (histogram and density curve (thick line) was significantly different from the distribution for similar samples from the full set of tumors estimated by permutation testing (thin line), with larger distances among tumors within an eye. C) The margin between tumors within an eye was estimated as the shortest distance between the boundaries of the tumor for comparison with the distance between the centroids of the tumors. D) The margin/distance ratio increased with distance, and the minimum ratio (~0.4) shows that the observed distribution of multiple tumors within an eye was not driven by close contact or crowding. (PDF) [file pone.0132932.s002.pdf]

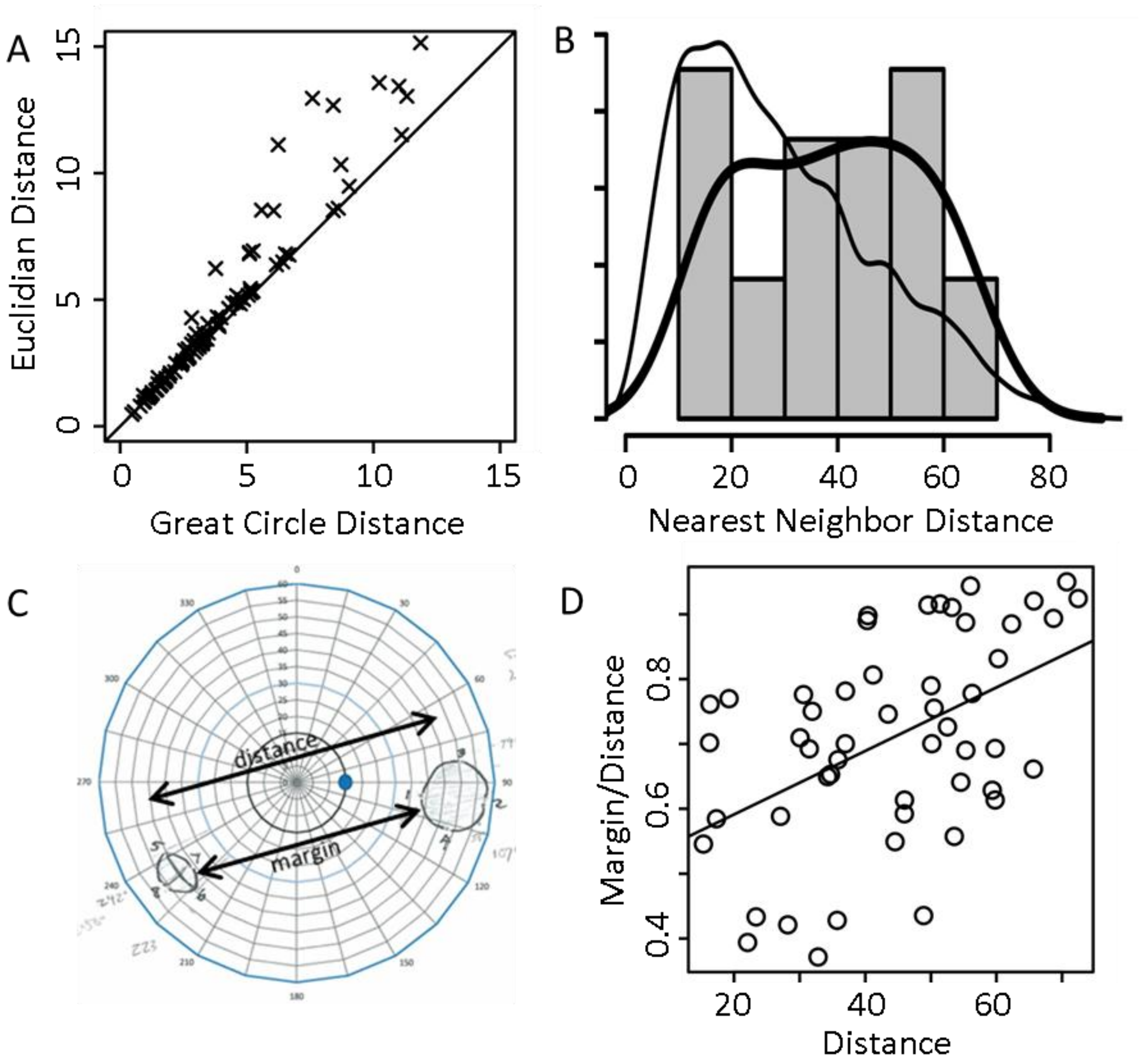

S3 Fig. A) Euclidian nearest neighbor distance in the azmuthal equidistant projection was greater than or equal to the actual great circle distance on the retinal surface, but the overall difference was not significant. B) The distribution of nearest neighbors distances for the subset of tumors in eyes containing multiple tumors (histogram and density curve (thick line) was significantly different from the distribution for similar samples from the full set of tumors estimated by permutation testing (thin line), with larger distances among tumors within an eye. C) The margin between tumors within an eye was estimated as the shortest distance between the boundaries of the tumor for comparison with the distance between the centroids of the tumors. D) The margin/distance ratio increased with distance, and the minimum ratio (~0.4) shows that the observed distribution of multiple tumors within an eye was not driven by close contact or crowding.
