# Supplementary material for: Spatiotemporal Patterns of Tumor Occurrence in Children with Intraocular Retinoblastoma
Source: PLoS One. 2015 Jul 31;10(7):e0132932. doi: 10.1371/journal.pone.0132932 (PMC4521796; doi:10.1371/journal.pone.0132932)
Supplement: S3 Fig — The plots include all mapped tumors and also the small tumors that were unmapped, but were located by quadrant from clinical fundoscopy reports. Tumors from right eyes are indicated with a + sign, tumors from left eyes are unmarked. Patient age at diagnosis is indicated above each plot, and the plots are presented in order of increasing age. (PDF) [file pone.0132932.s003.pdf]

S4 Fig. Tumor centroid plots by individual patient, shown in azimuthal equidistant projection corresponding to a right eye. The plots include all mapped tumors and also the small tumors that were unmapped, but were located by quadrant from clinical fundoscopy reports. Tumors from right eyes are indicated with a + sign, tumors from left eyes are unmarked. Patient age at diagnosis is indicated above each plot, and the plots are presented in order of increasing age.

Legend:

For mapped tumors, the symbols encode mutation type (shape), age quartile (color), and tumor area quartile (size).

mutation/laterality

● somatic/unilateral
▲ germline/bilateral
△ germline/unilateral

age (months)

■ 0.7 to 5.6
■ 5.6 to 8.8
■ 8.8 to 13.2
■ 13.2 to 65.5

area (%retina)

■ <2 µ
■ 2 to 12 S
■ 12 to 33 M
■ 33 to 73 L

Unmapped tumors are indicated by small open circles (o), plotted at eccentricity of 35 degrees for tumors inside the equator and at 55 degrees for tumors beyond the equator. The symbols are located at the midpoint of the quadrant or on the meridian, according to the location reported

Lines connect multiple tumors within and eye:

——— mapped to mapped

——— mapped to unmapped

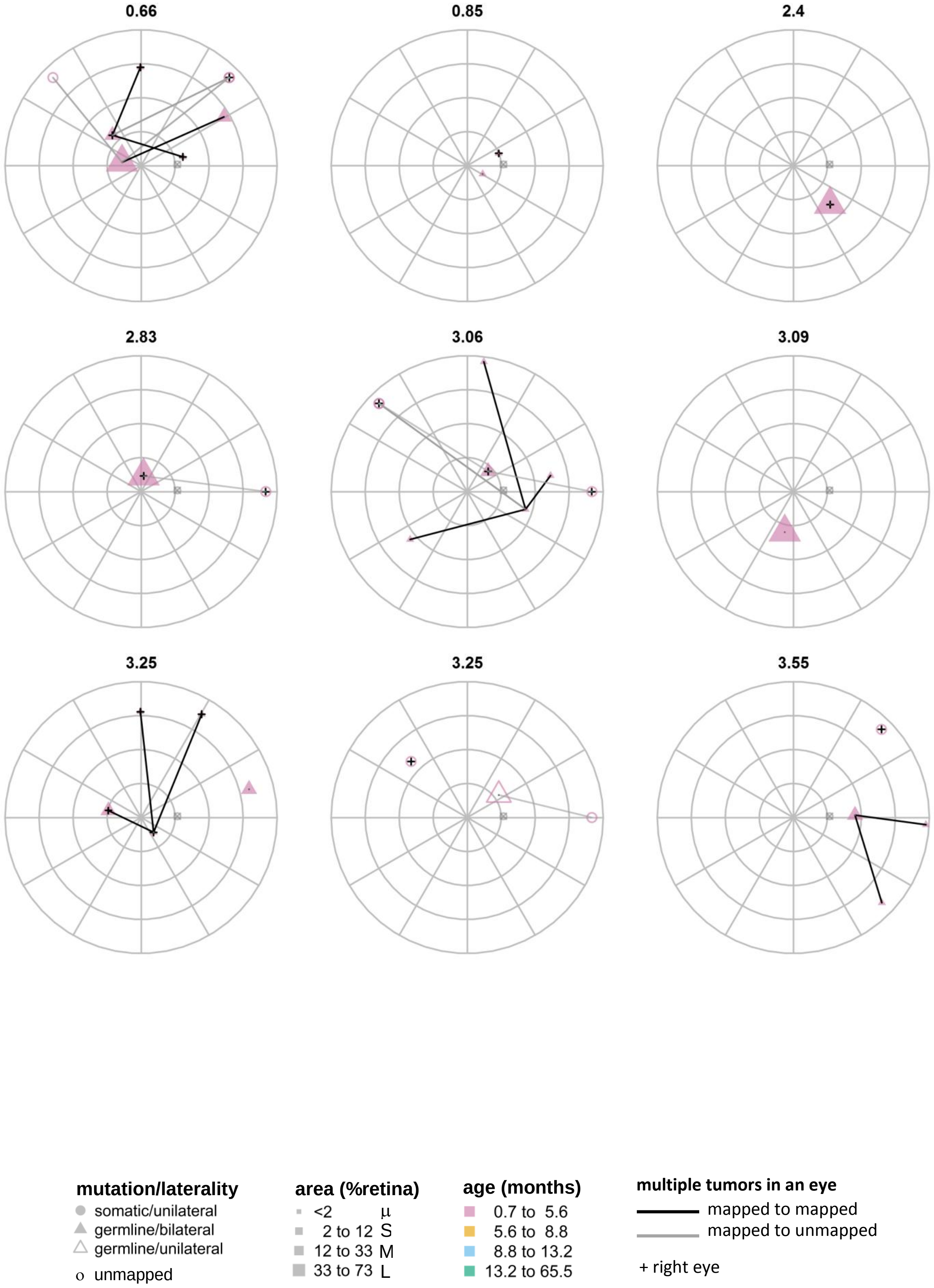
0.66
0.85
2.4
2.83
3.06
3.09
3.25
3.25
3.55
mutation/laterality
somatic/unilateral
germline/bilateral
germline/unilateral
o unmapped
area (%retina)
<2 μ
2 to 12 S
12 to 33 M
33 to 73 L
age (months)
0.7 to 5.6
5.6 to 8.8
8.8 to 13.2
13.2 to 65.5
multiple tumors in an eye
mapped to mapped
mapped to unmapped
+ right eye

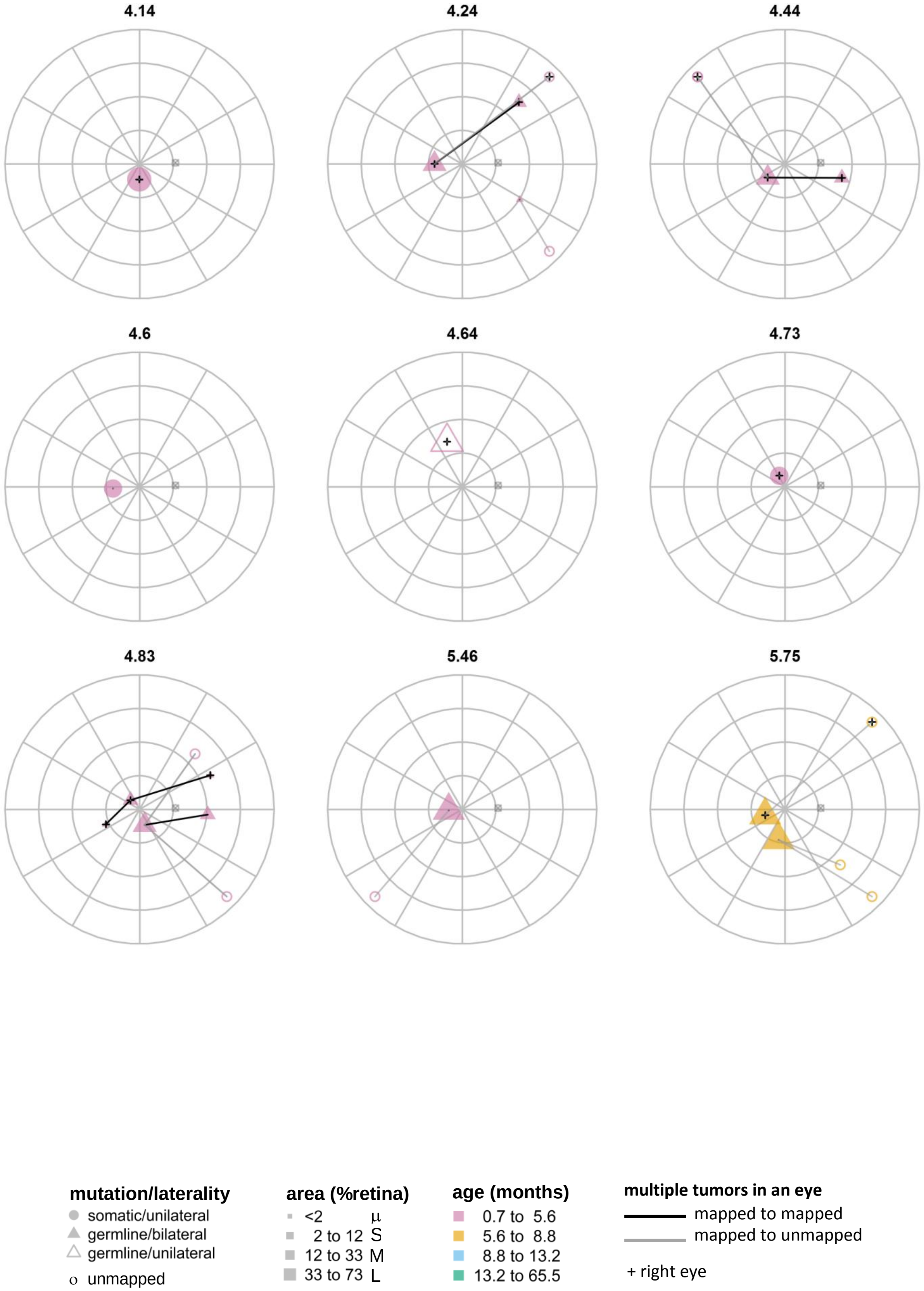
4.14
4.24
4.44
4.6
4.64
4.73
4.83
5.46
5.75
mutation/laterality
somatic/unilateral
germline/bilateral
germline/unilateral
unmapped
area (%retina)
<2 µ
2 to 12 S
12 to 33 M
33 to 73 L
age (months)
0.7 to 5.6
5.6 to 8.8
8.8 to 13.2
13.2 to 65.5
multiple tumors in an eye
mapped to mapped
mapped to unmapped
+ right eye

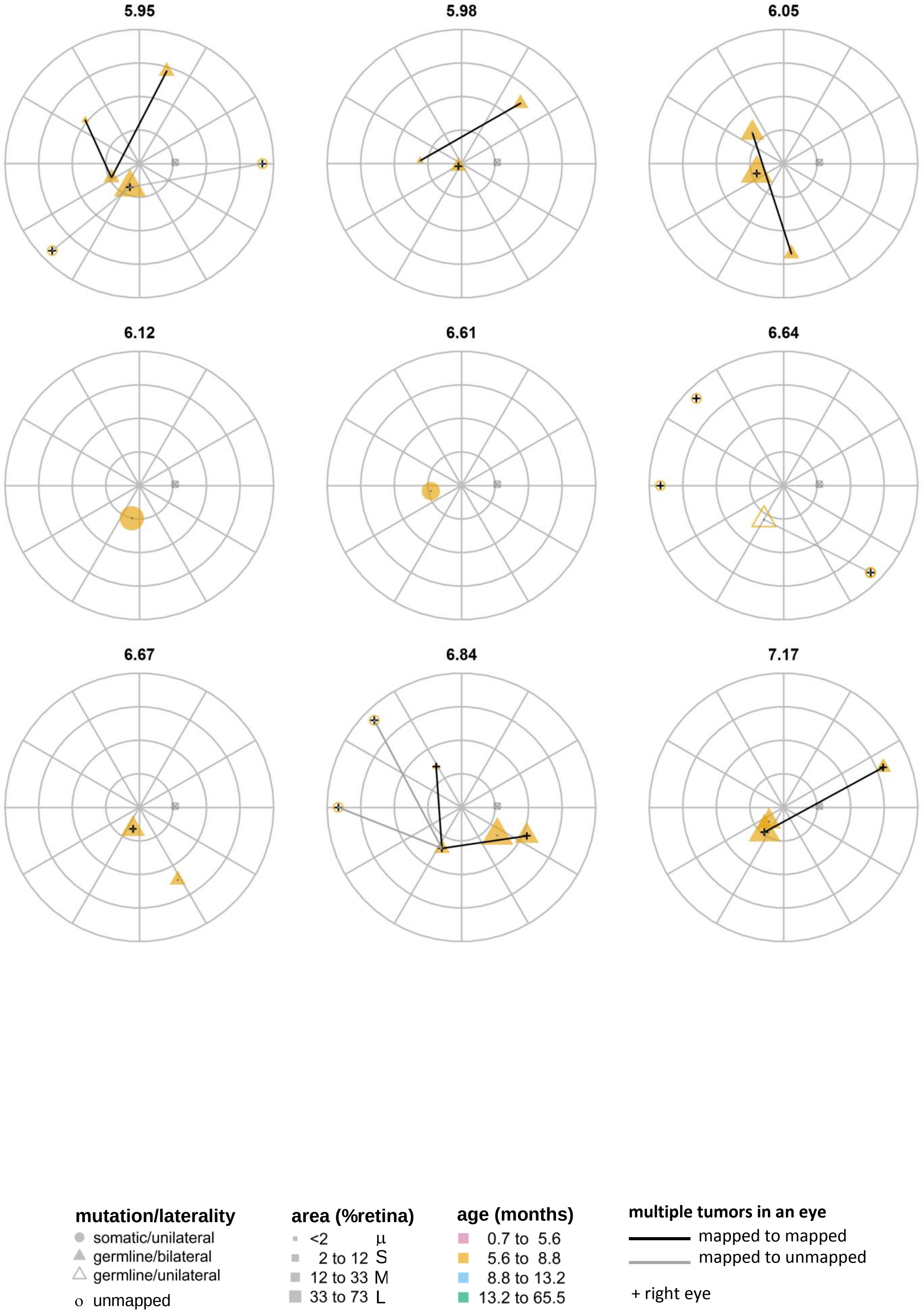
5.95
5.98
6.05
6.12
6.61
6.64
6.67
6.84
7.17
mutation/laterality
somatic/unilateral
germline/bilateral
germline/unilateral
o unmapped
area (%retina)
<2 µ
2 to 12 S
12 to 33 M
33 to 73 L
age (months)
0.7 to 5.6
5.6 to 8.8
8.8 to 13.2
13.2 to 65.5
multiple tumors in an eye
mapped to mapped
mapped to unmapped
+ right eye

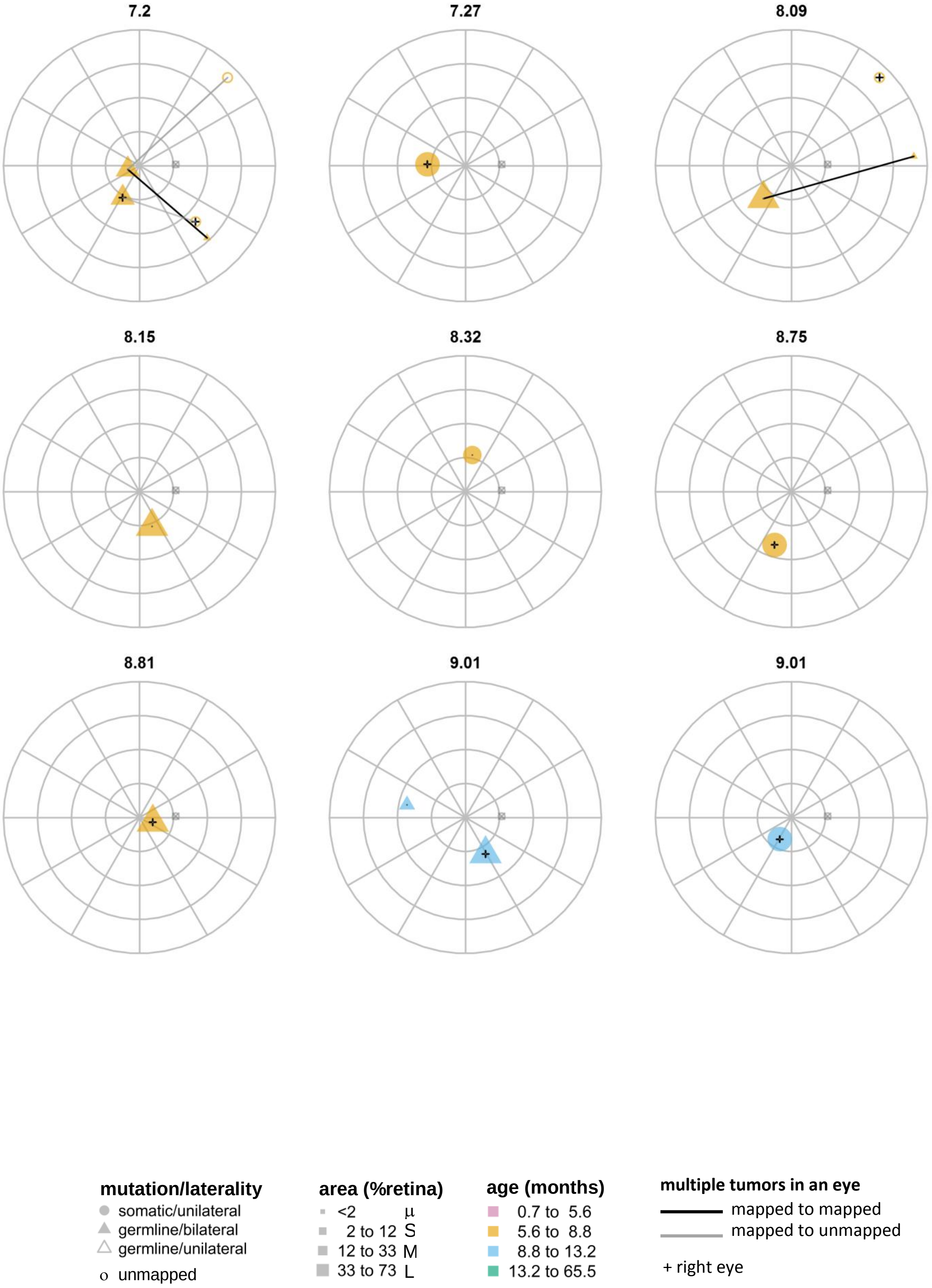
7.2
7.27
8.09
8.15
8.32
8.75
8.81
9.01
9.01
mutation/laterality
somatic/unilateral
germline/bilateral
germline/unilateral
o unmapped
area (%retina)
<2 μ
2 to 12 S
12 to 33 M
33 to 73 L
age (months)
0.7 to 5.6
5.6 to 8.8
8.8 to 13.2
13.2 to 65.5
multiple tumors in an eye
mapped to mapped
mapped to unmapped
+ right eye

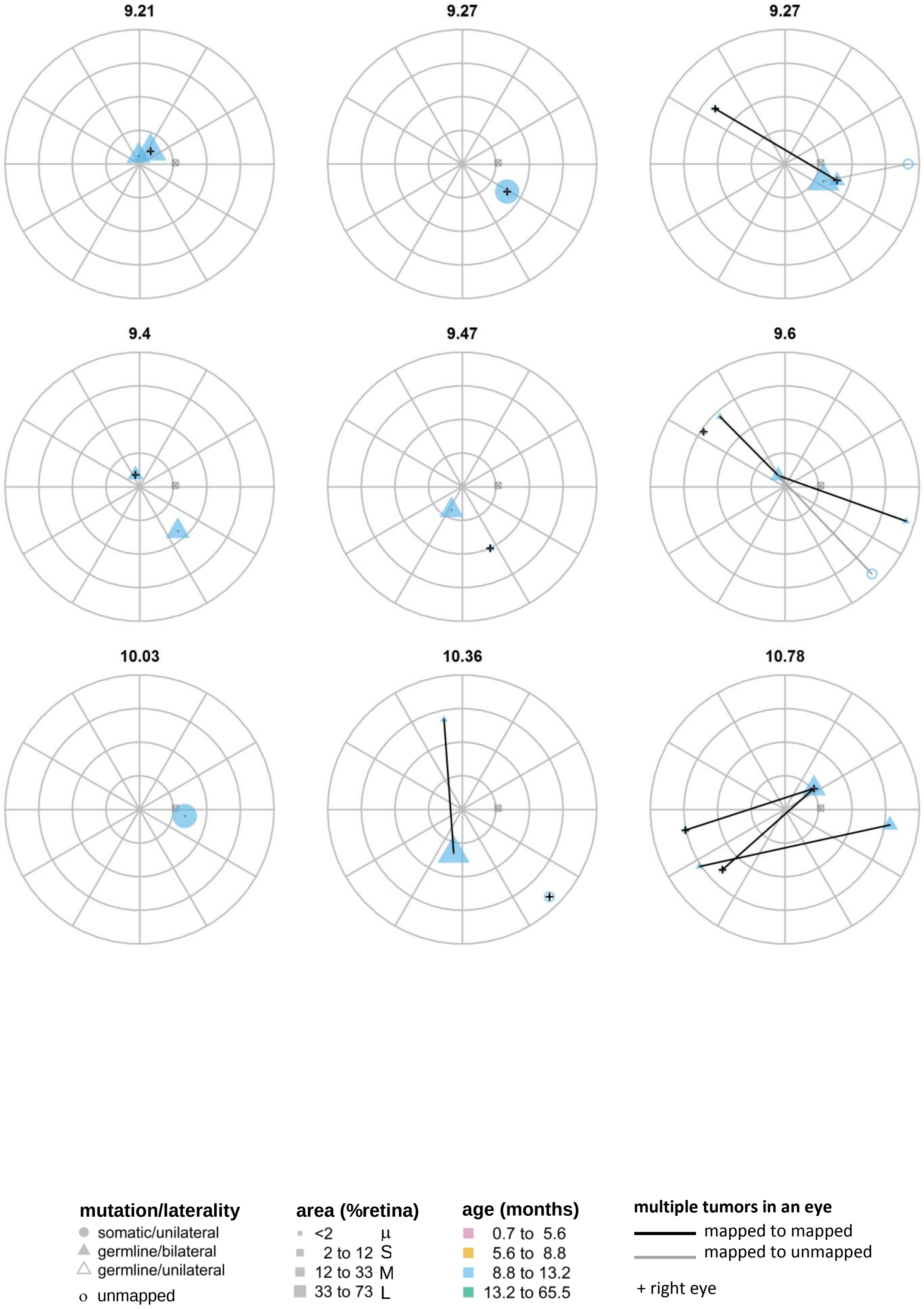
9.21
9.27
9.27
9.4
9.47
9.6
10.03
10.36
10.78
mutation/laterality
somatic/unilateral
germline/bilateral
germline/unilateral
o unmapped
area (%retina)
<2 µ
2 to 12 S
12 to 33 M
33 to 73 L
age (months)
0.7 to 5.6
5.6 to 8.8
8.8 to 13.2
13.2 to 65.5
multiple tumors in an eye
mapped to mapped
mapped to unmapped
+ right eye

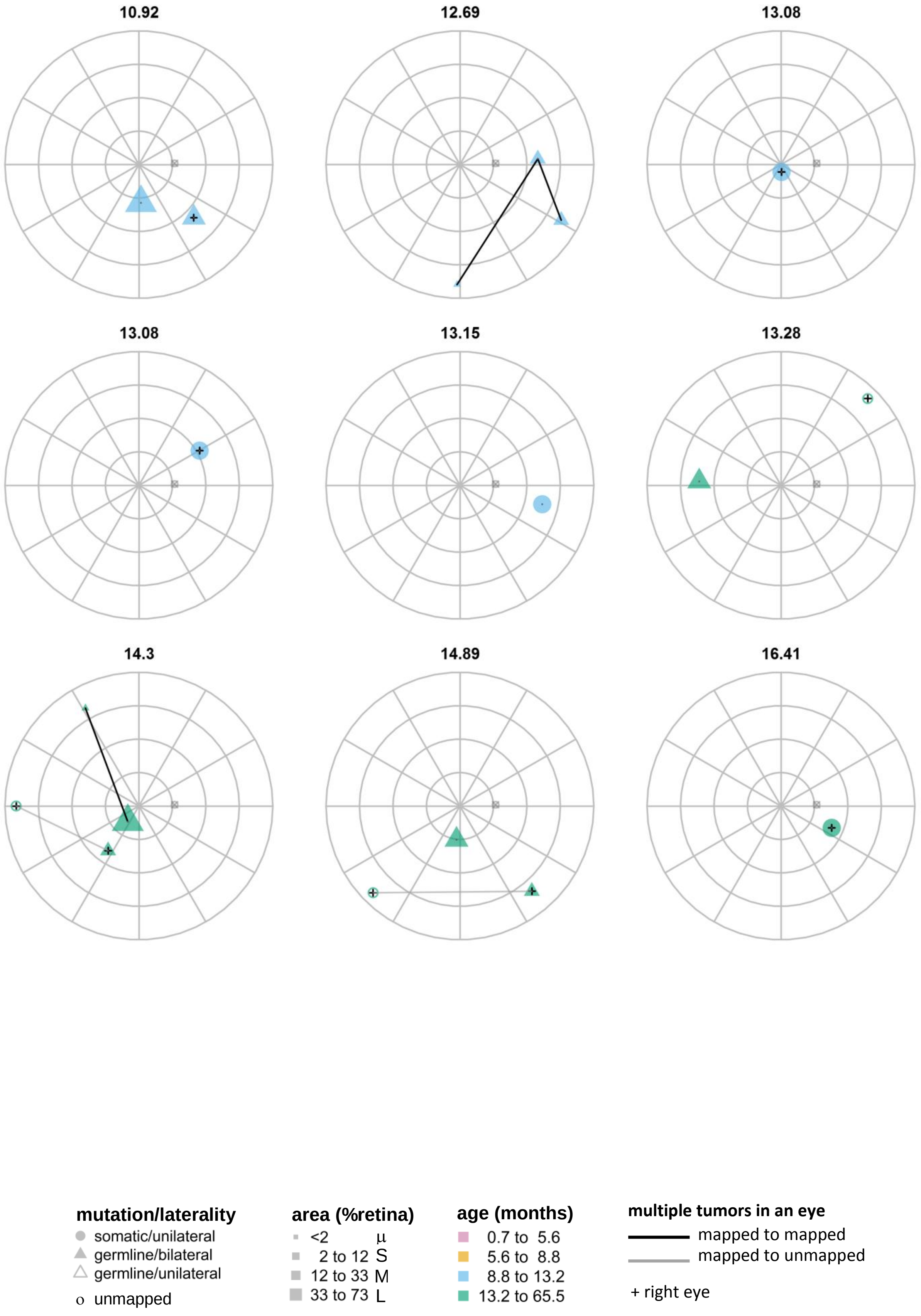
10.92
12.69
13.08
13.08
13.15
13.28
14.3
14.89
16.41
mutation/laterality
somatic/unilateral
germline/bilateral
germline/unilateral
o unmapped
area (%retina)
<2 µ
2 to 12 S
12 to 33 M
33 to 73 L
age (months)
0.7 to 5.6
5.6 to 8.8
8.8 to 13.2
13.2 to 65.5
multiple tumors in an eye
mapped to mapped
mapped to unmapped
+ right eye

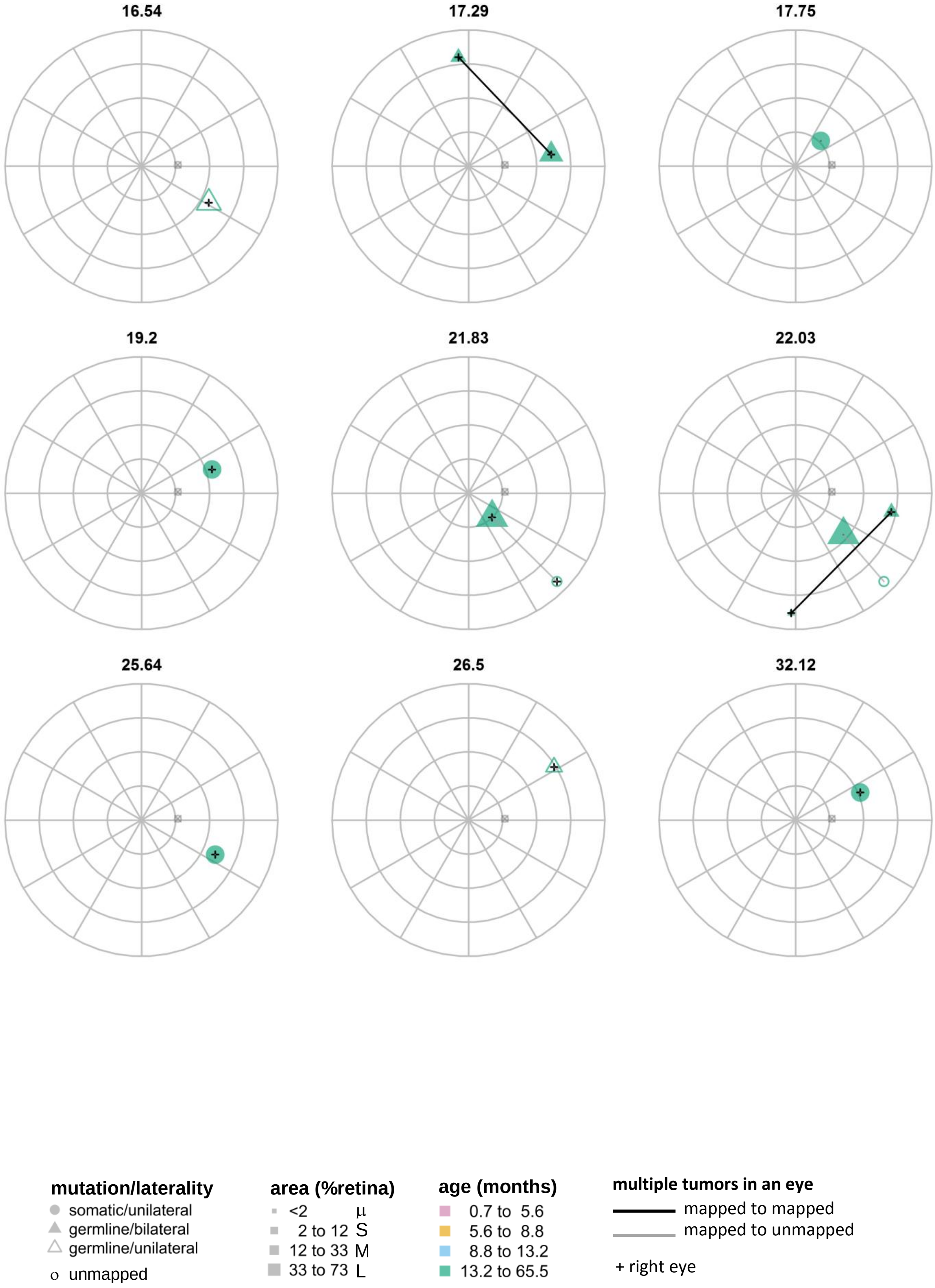
16.54
17.29
17.75
19.2
21.83
22.03
25.64
26.5
32.12
mutation/laterality
somatic/unilateral
germline/bilateral
germline/unilateral
o unmapped
area (%retina)
<2 μ
2 to 12 S
12 to 33 M
33 to 73 L
age (months)
0.7 to 5.6
5.6 to 8.8
8.8 to 13.2
13.2 to 65.5
multiple tumors in an eye
mapped to mapped
mapped to unmapped
+ right eye

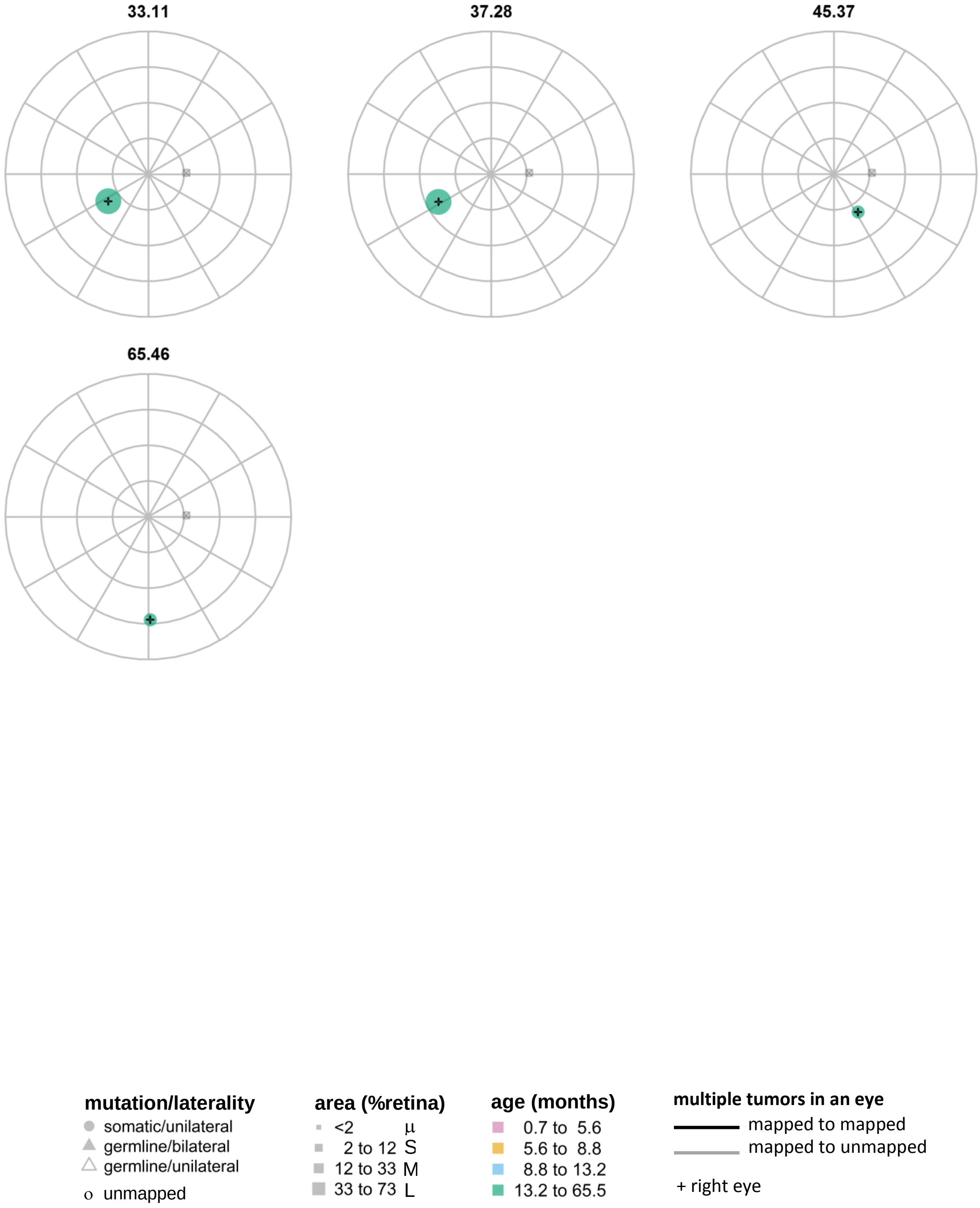
33.11
37.28
45.37
65.46
mutation/laterality
somatic/unilateral
germline/bilateral
germline/unilateral
o unmapped
area (%retina)
<2 µ
2 to 12 S
12 to 33 M
33 to 73 L
age (months)
0.7 to 5.6
5.6 to 8.8
8.8 to 13.2
13.2 to 65.5
multiple tumors in an eye
mapped to mapped
mapped to unmapped
+ right eye
