# Supplementary material for: Spatiotemporal Patterns of Tumor Occurrence in Children with Intraocular Retinoblastoma
Source: PLoS One. 2015 Jul 31;10(7):e0132932. doi: 10.1371/journal.pone.0132932 (PMC4521796; doi:10.1371/journal.pone.0132932)
Supplement: S1 Table — (PDF) [file pone.0132932.s004.pdf]

**S2 Table.** Poisson Point Process Models for distribution of tumor centroids marked by age at diagnosis quartile with polynomial covariates.

Model 1:

~marks + x + y + I(x^2) + I(y^2) + I(x^3) + I(x * y^2) + I(y^3) +

marks:x + marks:I(x^2) + marks:I(y^2) + marks:I(x^3) + marks:I(x * y^2) + marks:I(y^3)

Model 2:

~marks + x + y + I(x^2) + I(y^2) + I(x^3) + I(x * y^2) + I(y^3) + I(x^4) + I(x^2 * y^2) + I(x * y^3) +

marks:x + marks:I(x^2) + marks:I(y^2) + marks:I(x^3) + marks:I(x * y^2) + marks:I(y^3)

Model 3:

~marks + x + y + I(x^2) + I(y^2) + I(x^3) + I(x^2 * y) + I(x * y^2) + I(y^3) + I(x^4) + I(x^3 * y) + I(x^2 * y^2) + I(x * y^3) + I(x^5) + I(x^4 * y) + I(x^2 * y^3) + I(x * y^4) + I(y^5) +

marks:x + marks:y + marks:I(y^2) + marks:I(x^3) + marks:I(x^2 * y) + marks:I(x * y^2) + marks:I(y^3) + marks:I(x^4) + marks:I(x^3 * y) + marks:I(x^2 * y^2) + marks:I(x^5) + marks:I(x^4 * y) + marks:I(x * y^4)
Poisson

Model Comparison

| | Order | DOF | ΔDOF | Δdeviance | p | AIC |
|---|---|---|---|---|---|---|
| Model 1 | 3 | 29 | -- | -- | -- | 1645.5 |
| Model 2 | 4 | 32 | 3 | 9.2 | 0.0270 | 1642.3 |
| Model 3 | 5 | 60 | 28 | 58.6 | 0.0006 | 1639.7 |

*Order*: order of the covariate polynomial
*DOF*: degrees of freedom in the model
*ΔDOF*: difference in DOF from the model above in the table
*Δdeviance*: decrease in residual deviance compared to the model above in the table
*p*: p-value for Δdeviance
*AIC*: Akaike's Information Criterion

**See the following for additional details**. Baddeley, A. (2010) Analysing spatial point patterns in R. Workshop notes. Version 4.1. CSIRO online technical publication. URL: www.uwa.edu.au/resources/pf16h.html. Baddeley, A. and Turner, R. (2005a) Spatstat: an R package for analyzing spatial point patterns. Journal of Statistical Software 12:6, 1–42. URL: www.jstatsoft.org, ISSN: 1548-7660.
